# Sequential development of several RT‐qPCR tests using LNA nucleotides and dual probe technology to differentiate SARS‐CoV‐2 from influenza A and B

**DOI:** 10.1111/1751-7915.14031

**Published:** 2022-03-22

**Authors:** Monika Radvánszka, Evan D. Paul, Roman Hajdu, Kristína Boršová, Viera Kováčová, Piotr Putaj, Stanislava Bírová, Ivana Čirková, Martin Čarnecký, Katarína Buranovská, Adrián Szobi, Nina Vojtaššáková, Diana Drobná, Viktória Čabanová, Monika Sláviková, Martina Ličková, Veronika Vaňová, Sabína Fumačová Havlíková, Ľubomíra Lukáčiková, Ivana Kajanová, Juraj Koči, Diana Rusňáková, Tatiana Sedláčková, Klaas E. A. Max, Thomas Tuschl, Tomáš Szemes, Boris Klempa, Pavol Čekan

**Affiliations:** ^1^ 164047 MultiplexDX, s.r.o. Comenius University Science Park Ilkovičova 8 Bratislava 841 04 Slovakia; ^2^ MultiplexDX, Inc One Research Court, Suite 450 Rockville MD 20850 USA; ^3^ College of Medical, Veterinary and Life Sciences School of Life Sciences University of Glasgow University Avenue Glasgow G12 8QQ UK; ^4^ Biomedical Research Center Institute of Virology Slovak Academy of Sciences Dúbravská cesta 9 Bratislava 845 05 Slovakia; ^5^ Institute for Biological Physics University of Cologne Zülpicher Str. 77 Köln 50937 Germany; ^6^ 164047 Geneton s.r.o. Comenius University Science Park Ilkovičova 8 Bratislava 841 04 Slovakia; ^7^ Department of Molecular Biology Faculty of Natural Sciences Comenius University in Bratislava Ilkovičova 6 Bratislava 842 15 Slovakia; ^8^ 164047 Comenius University Science Park Ilkovičova 8 Bratislava 841 04 Slovakia; ^9^ 5929 Laboratory for RNA Molecular Biology The Rockefeller University 1230 York Avenue New York NY 10065 USA

## Abstract

Sensitive and accurate RT‐qPCR tests are the primary diagnostic tools to identify SARS‐CoV‐2‐infected patients. While many SARS‐CoV‐2 RT‐qPCR tests are available, there are significant differences in test sensitivity, workflow (e.g. hands‐on‐time), gene targets and other functionalities that users must consider. Several publicly available protocols shared by reference labs and public health authorities provide useful tools for SARS‐CoV‐2 diagnosis, but many have shortcomings related to sensitivity and laborious workflows. Here, we describe a series of SARS‐CoV‐2 RT‐qPCR tests that are originally based on the protocol targeting regions of the RNA‐dependent RNA polymerase (RdRp) and envelope (E) coding genes developed by the Charité Berlin. We redesigned the primers/probes, utilized locked nucleic acid nucleotides, incorporated dual probe technology and conducted extensive optimizations of reaction conditions to enhance the sensitivity and specificity of these tests. By incorporating an RNase P internal control and developing multiplexed assays for distinguishing SARS‐CoV‐2 and influenza A and B, we streamlined the workflow to provide quicker results and reduced consumable costs. Some of these tests use modified enzymes enabling the formulation of a room temperature‐stable master mix and lyophilized positive control, thus increasing the functionality of the test and eliminating cold chain shipping and storage. Moreover, a rapid, RNA extraction‐free version enables high sensitivity detection of SARS‐CoV‐2 in about an hour using minimally invasive, self‐collected gargle samples. These RT‐qPCR assays can easily be implemented in any diagnostic laboratory and can provide a powerful tool to detect SARS‐CoV‐2 and the most common seasonal influenzas during the vaccination phase of the pandemic.

## Graphical abstract

Here, we describe a series of SARS‐CoV‐2 RT‐qPCR tests that are originally based on the protocol targeting regions of the RNA‐dependent RNA polymerase (RdRp) and envelope (E) coding genes developed by the Charité Berlin. We redesigned the primers/probes, added an RNase P internal control, utilized locked nucleic acid nucleotides and dual probe technology to enhance sensitivity and specificity, optimized an RNA‐extraction free workflow, and multiplexed the assays to detect influenza. These RT‐qPCR assays can easily be implemented in any diagnostic laboratory and can provide a powerful tool to detect SARS‐CoV‐2 and the most common seasonal influenzas.

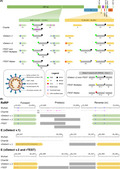

## Introduction

A comprehensive SARS‐CoV‐2 testing strategy is an important tool for countries to mitigate the spread of the coronavirus disease 2019 (COVID‐19) by facilitating early detection and implementation of appropriate epidemiological measures (Vandenberg *et al*., 2020; Weissleder *et al*., 2020). The gold standard for identifying SARS‐CoV‐2 entails using RT‐qPCR to detect the presence of one or more viral genes in a biological specimen. This method has unparalleled sensitivity, detecting down to single copies of viral RNA in a reaction, and can readily be deployed in diagnostic laboratories.

Shortly after the publication of the first SARS‐CoV‐2 genome sequences, several reference laboratories and public health authorities provided the first publicly available RT‐qPCR protocols (World Health Organization (WHO)). These protocols were instrumental in allowing countries to rapidly implement comprehensive testing strategies and often served as the backbone for commercial development of more streamlined tests with additional innovations. Currently, hundreds of RT‐qPCR tests have been developed to detect SARS‐CoV‐2 and studies comparing the efficacy of these tests revealed important differences in the specimen input, gene targets, testing workflow, specificity and sensitivity (Alcoba‐Florez *et al*., 2020; Nalla *et al*., 2020; van Kasteren *et al*., 2020; Vogels *et al*., 2020; Wang *et al*., 2020a).

The RT‐qPCR test developed by the Charité Institute of Virology in Berlin was the first protocol to be published (Corman *et al*., 2020) and shared by the WHO (World Health Organization (WHO)) and was widely used throughout Europe during the early stages of the pandemic. At the time of development, few SARS‐CoV‐2 sequences were publicly available and viral isolates and positive patient samples were scarce and unavailable for assay development; therefore, the authors designed an initial screening assay targeting the envelope (E) gene that intentionally cross‐reacts with SARS‐CoV viral RNA (from the 2003 outbreak) and a second confirmation assay targeting the RdRP gene contained two probes that differentiate SARS‐CoV‐2 from SARS‐CoV. The RdRP primers and SARS‐CoV‐2‐specific probe, however, contained several degenerate bases in areas thought to display genetic variability. The authors also pointed out that the design of the RdRP reverse primer could reduce reaction efficiency due to its low predicted melting temperature (Corman and Drosten, 2020). While this protocol provided unequivocal benefits in the early phase of diagnostic testing, a variety of issues emerged regarding the performance of this test, primarily reduced sensitivity of the RdRP assay (Etievant *et al*., 2020; Jung *et al*., 2020; Nalla *et al*., 2020; Pillonel *et al*., 2020; Vogels *et al*., 2020).

Here, we describe the development of several improved RT‐qPCR tests that address the limitations of the original Charité protocol and make significant strides in improving sensitivity, specificity and testing capabilities. We corrected base mismatches and optimized primer design using LNA‐modified nucleotides in our vDetect v.1 assay and incorporated an internal control (human RNase P) into our vDetect v.2 assay, creating a revamped version of the Charité protocol. By implementing technical innovations such as dual probe technology and a room temperature stable testing format in our rTest kit, we enhanced the sensitivity and specificity as well as eliminated the need for cold chain shipping and storage. In subsequent versions, we streamlined the workflow by multiplexing target assays in the rTest Multiplex and rTEST Allplex kits and developed an RNA extraction‐free rapid workflow using self‐collected gargle samples in the rTEST Rapid kit. Finally, in our rTEST COVID‐19/FLU kit, we conducted extensive bioinformatic analyses to develop additional assays to detect influenza A and B, providing a useful diagnostic tool to differentiate SARS‐CoV‐2 from the most common seasonal influenzas.

## Results

### Redesign and optimization of Charité SARS‐CoV‐2 E and RdRP primer/probe sets

**Fig. 1:**
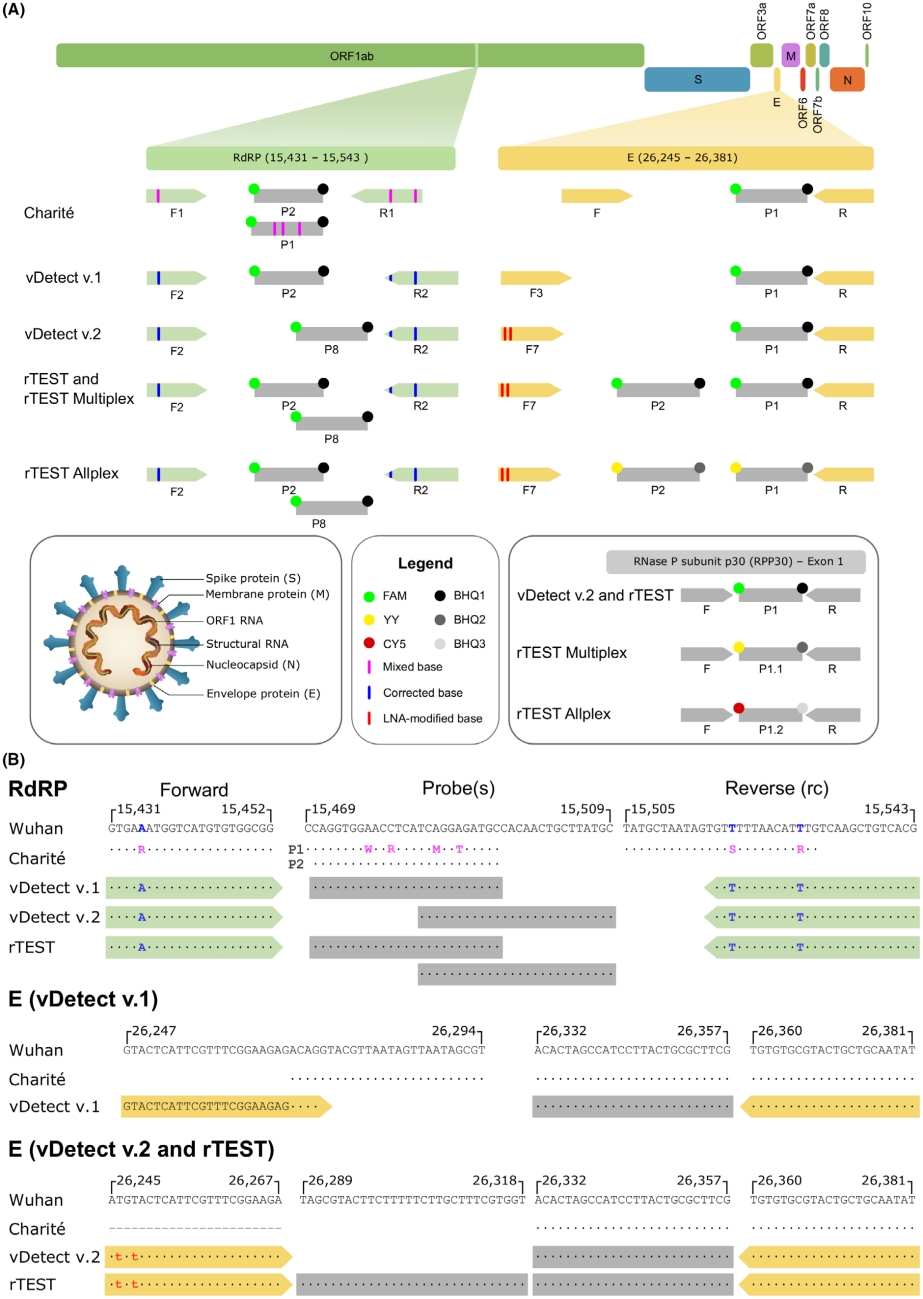
Schematic illustrating SARS‐CoV‐2 genome and regions targeted by RT‐qPCR primers and probes. A. Schematic overview portrays the SARS‐CoV‐2 genome with RdRP and E gene regions magnified to show the locations of primers and probes of the original Charité protocol, vDetect (v1 and v2), and rTEST RT‐qPCR assays. F, forward primer; P, probe; R, reverse primer. The inset boxes (from left to right) illustrate a SARS‐CoV‐2 particle with labelled structural proteins and RNA, legend describing panel A and the primers and probes used in each test to detect RNase P subunit p30 (RPP30). B. Diagram compares the sequences of RdRP and E gene primers and probes for the original Charité protocol, vDetect (v.1) and vDetect v.2 and rTEST RT‐qPCR assays to the Wuhan reference sequence. The numbers written above the Wuhan reference sequence correspond to the start and end base positions of the sequence Reverse primer sequences are written in the reverse complement (rc). Magenta lines and letters represent mixed bases found in the primers and probes in the Charité protocol that were replaced with the correct bases in vDetect v1 (blue lines and letters). Red lines and letters signify LNA‐modified bases.

As a starting point for our SARS‐CoV‐2 RT‐qPCR test, we used the E and RdRP primer/probe sets developed by the Charité Institute of Virology (Berlin) as a backbone for our test development. We aligned 505 SARS‐CoV‐2 sequences against the Wuhan reference genome and used the 95% consensus sequence to verify the specificity of the Sarbecco E gene and RdRP gene primer/probe set. Since the alignments identified several degenerate bases placed in the RdRP forward and reverse primers that resulted in mismatches to the consensus sequence, we replaced these degenerate bases with the appropriate complementary bases (Fig. 1).

**Fig. 2:**
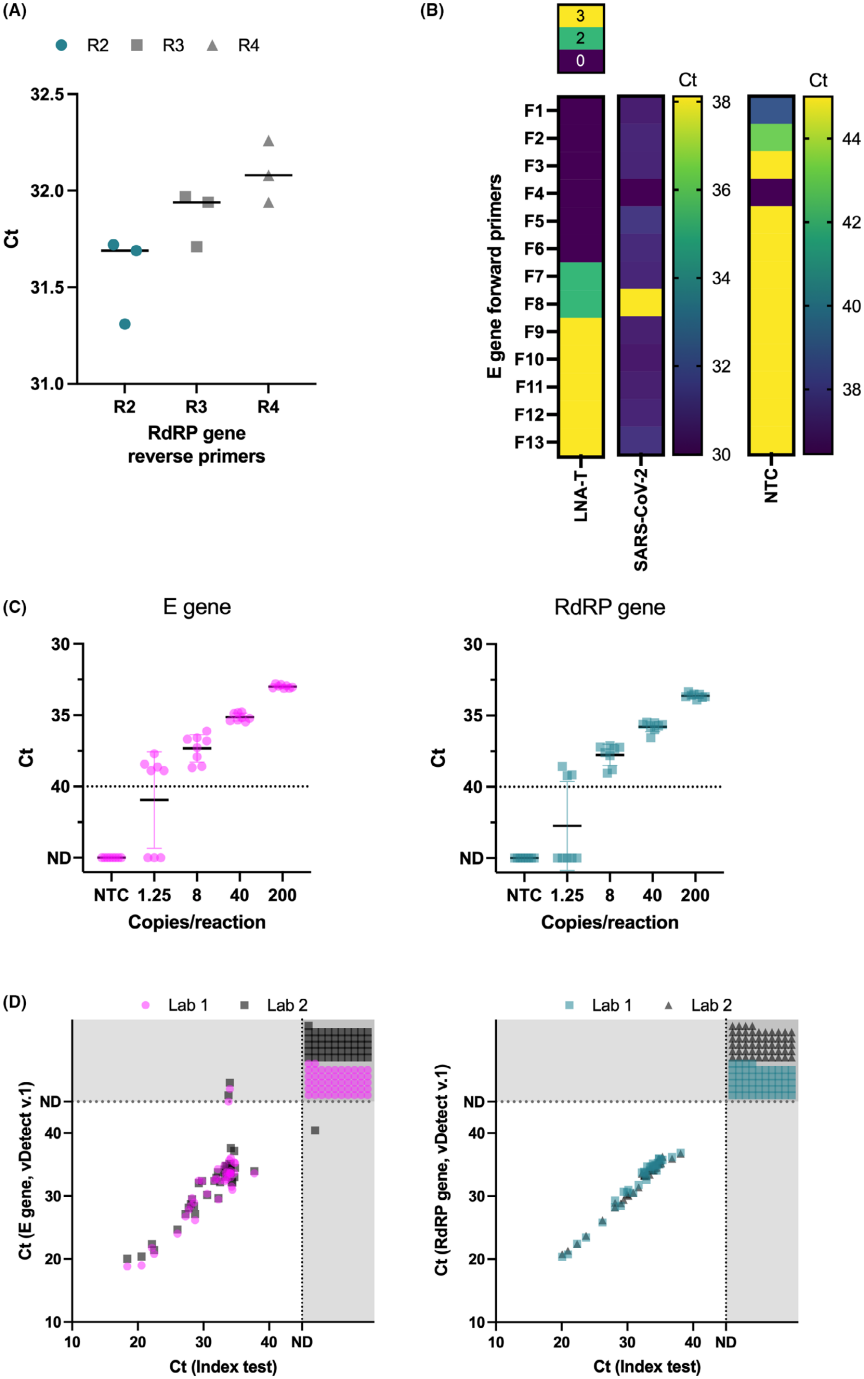
Redesign and validation of Charité SARS‐CoV‐2 E and RdRP primer/probe sets. A. Performance of redesigned RdRP gene reverse primers with replaced mixed bases and optimized melting temperatures. B. Heatmap shows various E gene forward primers with and without LNA‐modified thymine residues (LNA‐T) and their relative performance amplifying SARS‐CoV‐2 template or samples contaminated with E gene synthetic positive control. C. Limit of detection of both E (left panel) and RdRP (right panel) gene assays. Dotted line at *C*_t_ = 40 denotes the detection cut‐off. D. Clinical evaluation of both vDetect v.1 E (left panel) and RdRP (right panel) gene assays conducted in two independent laboratories. Dotted lines at ND and shaded areas show detection cut‐off and samples that were not detected for either the vDetect v.1 assay, index test assay, or both assays. *C*_t_, cycle threshold; E, envelope gene; ND, not detected within 45 cycles; NTC, no template control; R, reverse primer; RdRP, RNA‐dependent RNA polymerase.

An *in silico* analysis to assess melting temperatures and the potential formation of dimers and secondary structures also found that the RdRP reverse primer had a significantly lower annealing temperature relative to the forward primer (8.5°C Tm difference). This deficiency in primer design could reduce the efficiency and sensitivity of the RdRP assay. To address this, we designed three primers located downstream of the Charité reverse primer (R1), lengthened the primers by one base and incorporated LNA‐modified nucleotides to stabilize the 3’‐end and increase the melting temperatures of the forward primers (Table 1 and Table S1). While all primer sets amplified the SARS‐CoV‐2 positive control, the unmodified primer (R2) performed better than the LNA‐modified reverse primers (R3 and R4; B; Fig. 2A). This R2 reverse primer also had a closer melting temperature to the forward primer (3.3°C Tm difference) so we selected R2 as the reverse primer for further experiments.

**Table 1:** Primers and probes used for SARS‐CoV‐2, IAV and IBV detection.

Kit	Primer/probe name	Sequence (5′‐3′)	Conc. (nM)
vDetect v1	E_Sarbeco_F3	GTACTCATTCGTTTCGGAAGAGACAG	500
	E_Sarbeco_R2	ATATTGCAGCAGTACGCACACA	500
	E_Sarbeco_P1	FAM‐ACACTAGCCATCCTTACTGCGCTTCG‐ BHQ1	200
	RdRP_SARSr‐F2	GTGAAATGGTCATGTGTGGCGG	600
	RdRP_Delta‐F2	GTGAAATGGTCATGTGTGGC **A** G	600
	RdRP_SARSr‐R2	CGTGACAGCTTGACAAATGTTAAAAAC	800
	RdRP_SARSr‐P2	FAM‐CAGGTGGAACCTCATCAGGAGATGC‐BHQ1	200
vDetect v2	E_Sarbeco_F7	A**t**G**t**ACTCATTCGTTTCGGAAGA	500
	E_Sarbeco_R2	ATATTGCAGCAGTACGCACACA	500
	E_Sarbeco_P1	FAM‐ACACTAGCCATCCTTACTGCGCTTCG‐ BHQ1	200
	RdRP_SARSr‐F2	GTGAAATGGTCATGTGTGGCGG	600
	RdRP_Delta‐F2	GTGAAATGGTCATGTGTGGC **A** G	600
	RdRP_SARSr‐R2	CGTGACAGCTTGACAAATGTTAAAAAC	800
	RdRP_SARSr‐P8	FAM‐TCAGGAGATGCCACAACTGCTTATGC‐BHQ1	200
	RNase P Forward	AGATTTGGACCTGCGAGCG	500
	RNase P Reverse	GAGCGGCTGTCTCCACAAGT	500
	RNase P Probe 1	FAM‐TTCTGACCTGAAGGCTCTGCGCG‐BHQ1	160
rTEST	E_Sarbeco_F7	A**t**G**t**ACTCATTCGTTTCGGAAGA	500
	E_Sarbeco_R2	ATATTGCAGCAGTACGCACACA	500
	E_Sarbeco_P1	FAM‐ACACTAGCCATCCTTACTGCGCTTCG‐ BHQ1	200
	E_Sarbeco_P2	FAM‐TAGCGTACTTCTTTTTCTTGCTTTCGTGGT‐BHQ1	200
	RdRP_SARSr‐F2	GTGAAATGGTCATGTGTGGCGG	600
	RdRP_Delta‐F2	GTGAAATGGTCATGTGTGGC **A** G	600
	RdRP_SARSr‐R2	CGTGACAGCTTGACAAATGTTAAAAAC	800
	RdRP_SARSr‐P2	FAM‐CAGGTGGAACCTCATCAGGAGATGC‐BHQ1	200
	RdRP_SARSr‐P8	FAM‐TCAGGAGATGCCACAACTGCTTATGC‐BHQ1	200
	RNase P Forward	AGATTTGGACCTGCGAGCG	500
	RNase P Reverse	GAGCGGCTGTCTCCACAAGT	500
	RNase P Probe 1	FAM‐TTCTGACCTGAAGGCTCTGCGCG‐BHQ1	160
rTEST Multiplex	E_Sarbeco_F7	A**t**G**t**ACTCATTCGTTTCGGAAGA	500
	E_Sarbeco_R2	ATATTGCAGCAGTACGCACACA	500
	E_Sarbeco_P1	FAM‐ACACTAGCCATCCTTACTGCGCTTCG‐ BHQ1	187.5
	E_Sarbeco_P2	FAM‐TAGCGTACTTCTTTTTCTTGCTTTCGTGGT‐BHQ1	187.5
	RdRP_SARSr‐F2	GTGAAATGGTCATGTGTGGCGG	600
	RdRP_Delta‐F2	GTGAAATGGTCATGTGTGGC **A** G	600
	RdRP_SARSr‐R2	CGTGACAGCTTGACAAATGTTAAAAAC	800
	RdRP_SARSr‐P2	FAM‐CAGGTGGAACCTCATCAGGAGATGC‐BHQ1	187.5
	RdRP_SARSr‐P8	FAM‐TCAGGAGATGCCACAACTGCTTATGC‐BHQ1	187.5
	RNase P Forward	AGATTTGGACCTGCGAGCG	312.5
	RNase P Reverse	GAGCGGCTGTCTCCACAAGT	312.5
	RNase P Probe 1.1	YY‐TTCTGACCTGAAGGCTCTGCGCG‐BHQ2	87.5
rTEST Allplex and rTEST Rapid	E_Sarbeco_F7	A**t**G**t**ACTCATTCGTTTCGGAAGA	500
	E_Sarbeco_R2	ATATTGCAGCAGTACGCACACA	500
	E_Sarbeco_P1.2	YY‐ACACTAGCCATCCTTACTGCGCTTCG‐ BHQ2	200
	E_Sarbeco_P2.2	YY‐TAGCGTACTTCTTTTTCTTGCTTTCGTGGT‐BHQ2	200
	RdRP_SARSr‐F2	GTGAAATGGTCATGTGTGGCGG	600
	RdRP_Delta‐F2	GTGAAATGGTCATGTGTGGC **A** G	600
	RdRP_SARSr‐R2	CGTGACAGCTTGACAAATGTTAAAAAC	800
	RdRP_SARSr‐P2	FAM‐CAGGTGGAACCTCATCAGGAGATGC‐BHQ1	200
	RdRP_SARSr‐P8	FAM‐TCAGGAGATGCCACAACTGCTTATGC‐BHQ1	200
	RNase P Forward	AGATTTGGACCTGCGAGCG	250
	RNase P Reverse	GAGCGGCTGTCTCCACAAGT	250
	RNase P Probe 1.2	Cy5‐TTCTGACCTGAAGGCTCTGCGCG‐BHQ3	80
rTEST COVID‐19/Flu	E_Sarbeco_F7	A**t**G**t**ACTCATTCGTTTCGGAAGA	500
	E_Sarbeco_R2	ATATTGCAGCAGTACGCACACA	500
	E_Sarbeco_P1	FAM‐ACACTAGCCATCCTTACTGCGCTTCG‐ BHQ1	200
	E_Sarbeco_P2	FAM‐TAGCGTACTTCTTTTTCTTGCTTTCGTGGT‐BHQ1	200
	RdRP_SARSr‐F2	GTGAAATGGTCATGTGTGGCGG	600
	RdRP_Delta‐F2	GTGAAATGGTCATGTGTGGC **A** G	600
	RdRP_SARSr‐R2	CGTGACAGCTTGACAAATGTTAAAAAC	800
	RdRP_SARSr‐P2	FAM‐CAGGTGGAACCTCATCAGGAGATGC‐BHQ1	200
	RdRP_SARSr‐P8	FAM‐TCAGGAGATGCCACAACTGCTTATGC‐BHQ1	200
	RNase P Forward	AGATTTGGACCTGCGAGCG	250
	RNase P Reverse	GAGCGGCTGTCTCCACAAGT	250
	RNase P Probe 1.2	Cy5‐TTCTGACCTGAAGGCTCTGCGCG‐BHQ3	80
	IAV‐F1.1	TTCTAGCATGGTGGAGGCCAT	500
	IAV‐R1.2	CCGTCTGAGTTCTTCAATGGTGG	500
	IAV‐Probe1.2	YY‐TCTAGGGCCCGGATTGATGCCA‐BHQ2	200
	IBV‐F2.1	AGTGGACTCAGGAAAGTGGC	500
	IBV‐R2.3	TCCAT**t**TG**t**TGCATTGATTGAAGC	500
	IBV‐Probe2.3	YY‐TCC**a**AATGAA**a**TGGGGAATGGAAGCT‐BHQ2	200

Lowercase and bold letters represent LNA‐modified nucleotides. Red and bold letters represent mismatched base that was corrected for Delta variant.

At the beginning of the pandemic, labs worldwide implemented RT‐qPCR protocols to detect SARS‐CoV‐2 that were largely based on WHO‐approved protocols like the Charité assay. Because of the excessive demand for primers/probes and synthetic positive controls, many labs reported receiving primers/probes that were contaminated with synthetic positive controls for the E gene (Fischer *et al*., 2020; Huggett *et al*., 2020; Mögling *et al*., 2020; Wernike *et al*., 2021). We also experienced contaminated primer/probe sets with synthetic E gene templates so we redesigned the E gene forward primer by shifting its location to a more upstream location to create a primer/probe set that would not amplify the most common SARS‐CoV‐2 E gene synthetic controls (Fig. 1). Since the E gene is only 228 bases, we also incorporated varying numbers of LNA nucleotides enabling the design of shorter forward primers that do not overlap with the original Charité forward primer, while maintaining sufficient melting temperatures. While all these primers amplified SARS‐CoV‐2 template equally well, except for F8, some of the forward primers that overlapped with the original Charité forward primer continued to display amplification in the absence of template (e.g. F1, F2 and F4; Fig. 2B). Based on these data as well as the fluorescent intensity generated by each primer/probe set, we selected F3 as the optimal forward primer (Fig. 1). We also investigated the optimal reverse transcription (RT) and annealing temperatures. Temperatures deviating above or below the standard RT (50°C) were either detrimental or had no effect on amplification for either E or RdRP assays. Although a lower annealing temperature (58°C) caused minor improvement in E gene detection, we opted to maintain the manufacturer’s recommendations (Fig. S1A).

With this final primer/probe set, called vDetect v.1, we determined the LoD for both E and RdRP assays to be 8 copies/reaction (Fig. 2C) and conducted cross‐reactivity tests to other closely related coronaviruses, revealing high specificity since the E and RdRP assays did not amplify other coronaviruses (Table S3). To assess the clinical performance of this revamped version of the Charité protocol, called vDetect v.1 COVID‐19 RT‐qPCR test, two independent labs conducted the test on 92 clinical samples and compared the results to the E and RdRP assays from the original Charité protocol that was used for routine SARS‐CoV‐2 screening by public health officials. Like the original Charité protocol, the test workflow consisted of an initial screening test for detection of the E gene followed by a confirmation test for detection of the RdRP gene. Our vDetect v.1 test correctly identified all positive (38/38) and negative (52) samples, and even identified two false positive samples that were incorrectly classified by the Charité E gene assay of the reference method (Fig. 2D; Table S4).

Since the LoD of vDetect v.1 was slightly less sensitive than the reported LoDs for E and RdRP (5.2 and 3.8 copies/reaction respectively) in the original Charité protocol (Corman *et al*., 2020), we switched to the Agilent Brilliant III Ultra‐Fast QRT‐PCR Master Mix, which in our internal testing yielded superior results, and conducted a thorough optimization of reaction parameters and reaction composition. Initially, we assessed the performance of a variety of parameters by analysing PCR products using gel electrophoresis and found that extending the RT reaction to 30 min and increasing the initial denaturation temperature to 97°C were beneficial (Fig. S1B). To verify these beneficial modifications, we tested three thermal profile variants using RT‐qPCR and determined that extending the RT reaction to 30 min improved detection of RdRP and that elevating the initial denaturation temperature provided no additional benefit (Fig. S1C). Using RT‐qPCR, we also observed that increasing the concentration of reverse transcriptase (from 1.0 μl to either 1.1 or 1.5 μl) in the reaction either had no effect or was detrimental (Fig. S1D). Altogether, we decided to eliminate the use of DTT in the RT step and increased the RT time to 30 min.

We also modified the oligonucleotides used in the RdRP and E gene assays. First, we replaced the RdRP probe (P2) with a new TaqMan hydrolysis probe (P8) that resulted in a substantial increase in fluorescent intensity (Fig. 1). Also, after experiencing sporadic amplification of NTCs with our modified E gene assay that was due to contamination with synthetic E gene positive control, we replaced the E gene forward primer (F3) with a shorter LNA‐modified forward primer (F7) that did not overlap with the original forward primer and therefore eliminated amplification of the JRC synthetic positive control. With this new master mix, optimized reaction parameters, and modified RdRP and E gene primer/probe sets, we incorporated the US CDC human RNase P primer/probe (Centers for Disease Control and Prevention, 2020) as an internal control for RNA extraction and assay performance. With this new version, vDetect v2., we observed an improvement in sensitivity with both E and RdRP assays consistently detecting down to only 2 copies of viral RNA per reaction (Fig. S1E).

### Optimization of a room‐temperature stable SARS‐CoV‐2 RT‐qPCR assay

**Fig. 3:**
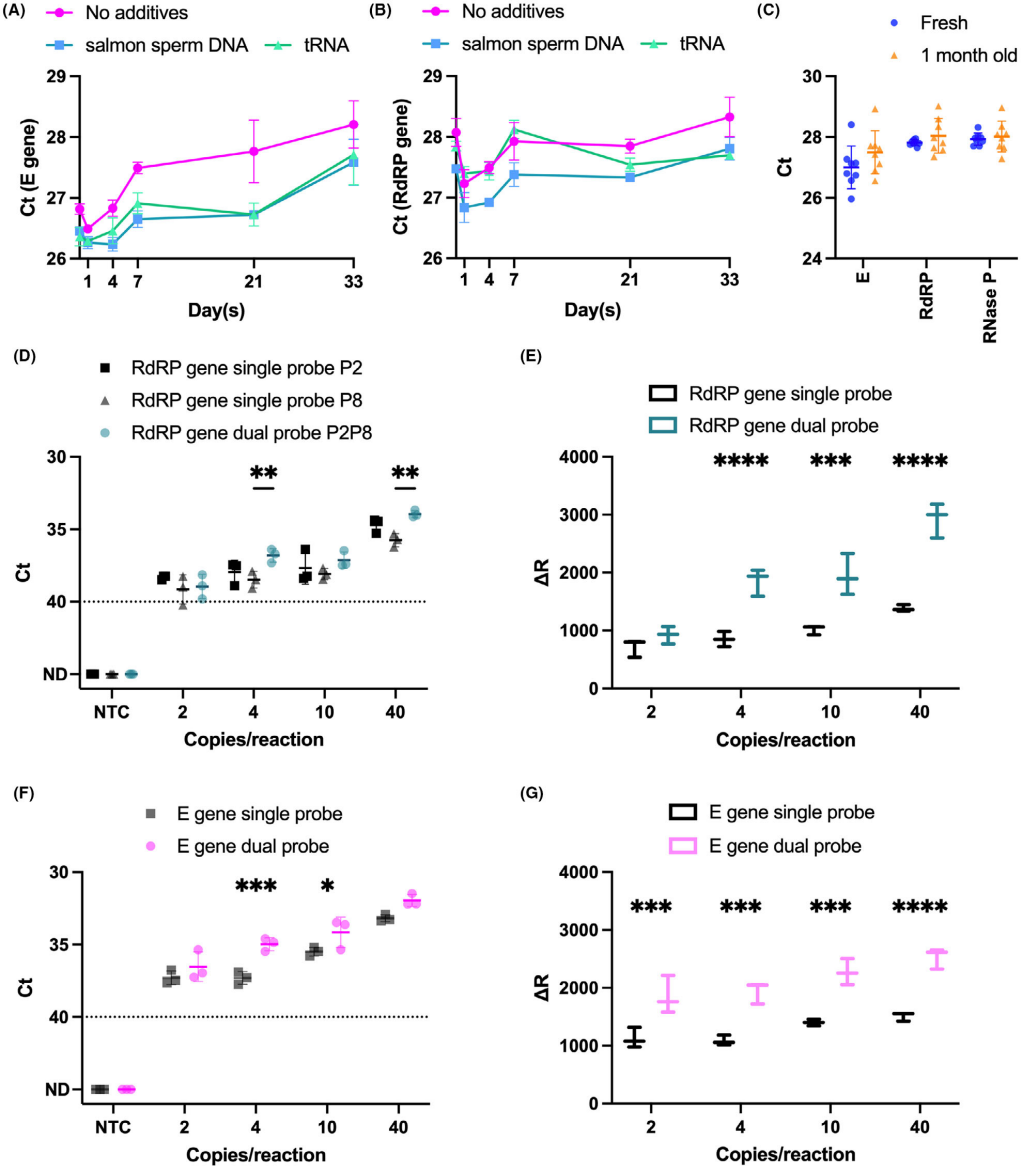
Optimization of room temperature stable kit and dual probes for rTEST COVID‐19 qPCR kit. (A, B) Graphs depict the effects of decoy nucleic acids (tRNA or salmon sperm DNA) or pure oligonucleotides (pure) on the stability of lyophilized positive control left at room temperature over a one‐month period as assessed by amplification of RdRP (A) and E (B) genes. (C) Performance of either fresh or an rTEST COVID‐19 qPCR kit left at room temperature for 1‐month on amplification of SARS‐CoV‐2 E and RdRP genes and human RNase P. Comparison of analytical sensitivity (D, F) and fluorescent intensity (E, G) between single probes versus dual probes for both RdRP (D, E) and E (F, G) genes. Dotted line at *C*_t_ = 40 serves as a threshold after which amplification is considered invalid. *P < 0.05, **P < 0.01, ***P < 0.001, ****P < 0.0001. *C*_t_, cycle threshold; E, envelope gene; ND, not detected within 45 cycles; NTC, no template control; P, probe; RdRP, RNA‐dependent RNA polymerase; tRNA, baker’s yeast transfer RNA; Δ*R*, normalized fluorescent intensity.

A major limitation of the majority of SARS‐CoV‐2 RT‐qPCR assays is the requirement to ship and store reaction components at low temperatures (−20°C). To address this disadvantage, we optimized our assay to be compatible with a room‐temperature stable master mix (SOLIScript^®^ 1‐step CoV Kit; SOLIS BioDyne), which contains genetically modified enzymes that possess a stability TAG that enhances their tolerance to a range of temperatures, stabilized and protected the lyophilized positive control using decoy nucleic acids, and conducted stability tests on the positive control and whole kit over a one‐month period. Using both gel electrophoresis of PCR products and real time RT‐qPCR, we found most modifications to the standard thermal cycling procedure produced no change or were detrimental (Fig. S2A and B), suggesting the standard reaction parameters were optimal for our assays. Since SOLIS BioDyne already verified the room temperature stability of the master mix, we focused our efforts on stabilizing and lyophilizing our positive control (PC BMC 5). Since the main source of RNA degradation at room temperature is due to nuclease activity, we spiked our positive control with either baker’s yeast tRNA or salmon sperm DNA to act as carriers and decoys for nucleases, and then lyophilized the positive control and tested its stability over a month period. Although all lyophilized positive controls, regardless of stabilizer, showed similar trends in *C*_t_ value over a one‐month period, the mean *C*_t_ values for samples containing either tRNA or salmon sperm DNA were generally lower than those for control samples containing no additives (Fig. 3A and B). Moreover, given that the relative increase in *C*_t_ values over a one‐month period was reduced for both tRNA and salmon sperm DNA, relative to the lyophilized control with no additives, we decided to spike our positive control with baker’s yeast tRNA. Leaving the entire kit (master mix, lyophilized primer/probe sets and positive control) at room temperature for one month and comparing performance to a freshly prepared kit demonstrated consistent performance for all assay targets for at least one month at room temperature (Fig. 3C).

### Dual probes enhance specificity and increase fluorescent signal

Although coronaviruses such as SARS‐CoV‐2 display reduced mutation rates relative to other RNA‐based viruses (Callaway, 2020; Lauring and Hodcroft, 2021), there is considerable evidence that emerging SARS‐CoV‐2 mutations can lead to increased transmissibility, virulence and escape from immune responses (Lauring and Hodcroft, 2021; Rahimi *et al*., 2021; Vilar and Isom, 2021). If mutations occur in diagnostic targets of RT‐qPCR assays, they can lead to reduced binding efficiency of primers and probes and consequently reduced sensitivity and even failed tests (Artesi *et al*., 2020; Jaroszewski *et al*., 2020; Khan and Cheung, 2020; Wang *et al*., 2020b). To address this issue, we designed a series of additional hydrolysis probes for both the E and RdRP assays that contain the same fluorescent reporter dyes as the first probe, essentially making the assays more robust to potential mutations in complementary sequences. Other groups have shown this dual probe approach increases the sensitivity and specificity of probe‐based RT‐qPCR assays (Yip *et al*., 2005; Nagy *et al*., 2017). In parallel, we also tested probes containing a second BHQ‐1 quencher located internally, which is reported to reduce background fluorescence and increase the dynamic range of the fluorogenic probes (Wilson *et al*., 2011; Hirotsu *et al*., 2020).

We screened a variety of RdRP probes with or without an internal quencher and surprisingly found that the internal quencher reduced sensitivity (i.e. increased *C*_t_) and had variable effects on fluorescent intensity (Fig. S2C). The best performing probe (P8, lowest *C*_t_ and highest fluorescence (Δ*R*)) when included in the same reaction as our original probe (P2) increased the sensitivity of the reaction (Fig. 3D) and drastically improved the dynamic range of the assay (Fig. 3E). It is noteworthy, that P2 and P8 slightly overlap on the same strand yet still produce a significant increase in sensitivity and fluorescence intensity, suggesting little, if any, interference between these overlapping probes.

For the E gene assay, we designed an additional probe (P2) downstream of the original probe P1 with two LNA‐modified variants (P3 and P4) as well as a probe (reverse complement of P1, P1rev), that would bind to the reverse strand located downstream of the reverse primer. LNA‐modified probes showed equivalent sensitivity to an unmodified probe (Fig. S2D). While a second probe complementary to the opposite strand was detrimental to sensitivity in both single and dual probe reactions (Fig. S2D), inclusion of a second, non‐overlapping probe in tandem with the original probe enhanced sensitivity of the assay (Fig. S2D). Indeed, like the RdRP assay, dual‐ versus single‐probe reactions increased sensitivity (Fig. 3F) and dynamic range (Fig. 3G).

With our optimized room‐temperature stable master mix and dual‐probe assays for SARS‐CoV‐2 E and RdRP genes, we assessed the LoD of this new test (called rTEST COVID‐19 qPCR) and confirmed an LoD of 2 copies/reaction (Fig. S3A). These modified E and RdRP primer/probe assays were also highly specific as there was no cross‐reactivity to a panel of closely related coronaviruses and other respiratory pathogens (Table S3). To investigate the clinical utility of this improved test, we thawed and re‐extracted RNA from a panel of 92 clinical samples and compared the rTEST COVID‐19 qPCR kit with an index test (vDetect COVID‐19 qPCR v2 kit). The index test failed to identify one true positive sample with the E gene assay and five true positive samples with the RdRP assay, likely due to loss of RNA during the freeze/thaw process or RNA extraction or both steps in the workflow; notably, both E and RdRP assays of the rTEST COVID‐19 qPCR kit correctly identified all negative (54/54, 100% diagnostic specificity) and positive (38/38, 100% diagnostic sensitivity) samples, including the samples that were positive in the original reference test, but negative in the retest of RNA extracted from thawed samples (Fig. S3B and C; Table S5).

The clinical performance of the rTEST COVID‐19 qPCR kit was also evaluated by an independent laboratory at Rockefeller University using 15 SARS‐CoV‐2 positive and 15 SARS‐CoV‐2 negative samples as determined by the index text (US CDC 2019‐nCoV Kit). This evaluation revealed high concordance between the rTEST COVID‐19 qPCR kit and the index test (US CDC 2019‐nCoV Kit) with both the E and RdRP genes assays of rTEST correctly identifying all positive and negative samples (Fig. S3D). Notably, the US CDC test produced a substantial amount of non‐specific amplification products with 7/15 (for N2 assay) and 3/15 (for N1 assay) negative samples amplifying between 38 (the cut‐off for positive samples) and 45 cycles. In contrast, rTEST displayed superior performance with only 2/30 negative samples (both RdRP assay) displaying non‐specific amplification after 38 cycles.

### Multiplexing E and RdRP gene assays to streamline testing workflow

Like the original Charité protocol, the workflow of our optimized test consisted of an initial screening test using the E gene and a second confirmation test for the RdRP gene. An additional third assay for the human RNase P internal control could be run in parallel. The shortcomings of this lengthy workflow are counterproductive given diagnostic labs may face significant backlogs in testing. To address this limitation and enable rapid, high throughput testing, we streamlined our test by multiplexing assay targets into a single reaction. We first multiplexed each of the E and RdRP gene assays (FAM dyes) with human RNase P (HEX dye). Given that two primer/probe sets were competing for a limited pool of reagents in a single reaction, we reduced the concentration of primers and probe for the more abundant RNase P assay to ensure this reaction would plateau before consuming all the reagents. We determined that that this primer limited multiplexed assay did not change the LoD (Fig. S4A). Indeed, this multiplexed test (called rTEST COVID‐19 qPCR Multiplex Kit), like its singleplexed counterpart, showed an impressive 100% diagnostic specificity (30/30 negative samples) and sensitivity (30/30 positive samples; Fig. S4B; Table S6). Several independent laboratories also validated the performance of this test, including the Centre for Infectious Diseases Research, Diagnostics and Laboratory Surveillance, National Institute for Health and Environment (Bilthoven, the Netherlands), Biomedical Center Martin, Comenius University in Bratislava, Jessenius Faculty of Medicine in Martin (Martin, Slovakia) and AnalytX, s.r.o. (Bratislava, Slovakia).

**Fig. 4:**
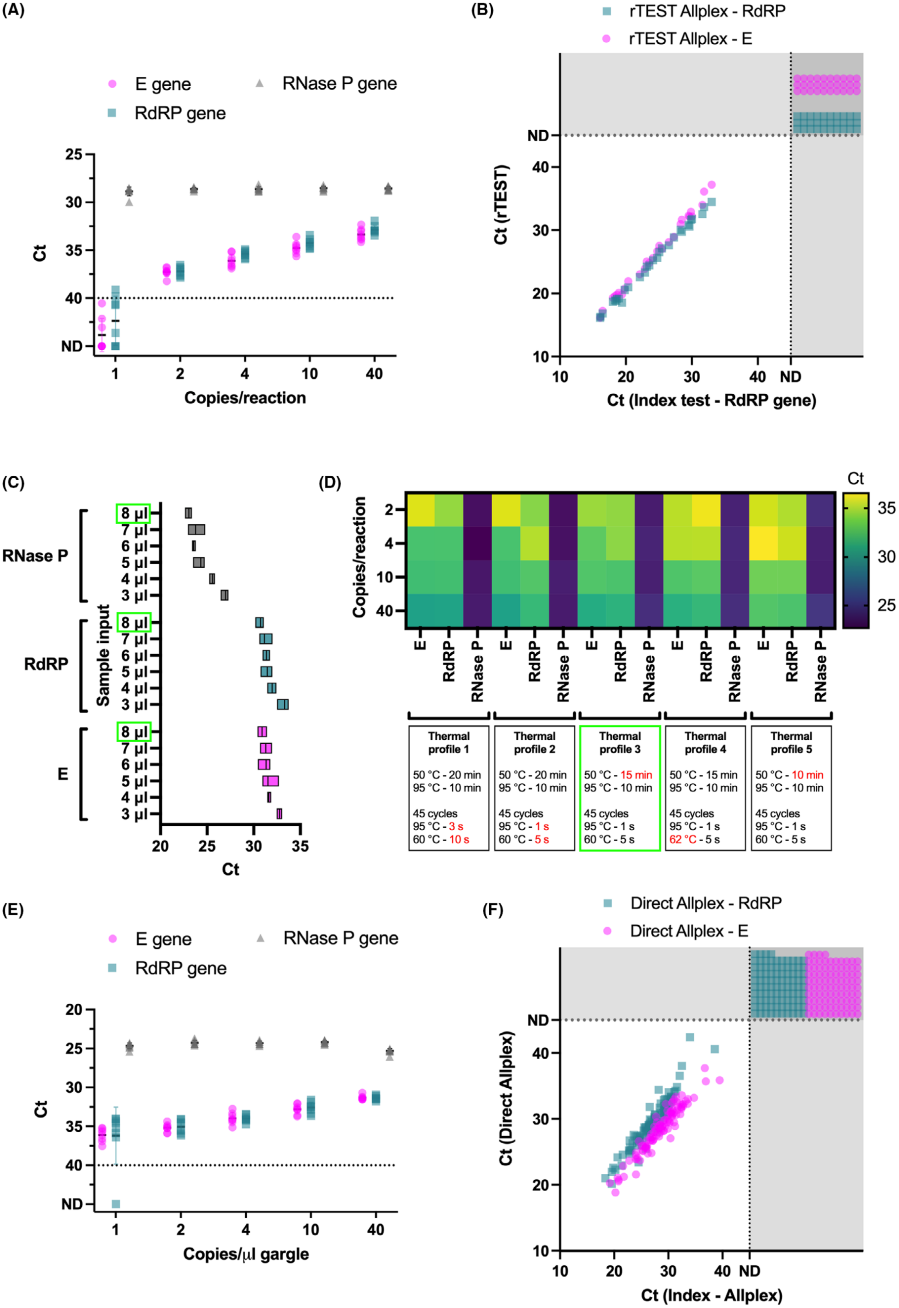
Optimization, analytical sensitivity and clinical performance of a rapid, RNA extraction‐free, multiplexed RT‐qPCR assay. A. Analytical sensitivity of the triplexed E, RdRP and RNase P assay in the rTEST COVID‐19 qPCR Allplex kit. B. Clinical performance of the rTEST COVID‐19 qPCR Allplex kit. C. Optimization of gargle sample input for a rapid, RNA extraction‐free, triplexed rTEST. D. Comparison of four different thermal profiles using 8 μl of gargle input volume in rapid, direct RT‐qPCR. E. Analytical sensitivity of the triplexed E, RdRP and RNase P assay in the RNA extraction‐free rTEST COVID‐19 qPCR Rapid kit. F. Clinical performance of the rTEST COVID‐19 qPCR Rapid kit. The dotted line at *C*_t_ = 40 (panels A and E) serves as a threshold after which amplification is considered invalid. The dotted lines and shaded areas (panels B and F) indicate samples that were not detected by either the evaluation test, index test or both tests. *C*_t_, cycle threshold; E, envelope gene; ND, not detected within 45 cycles; NTC, no template control; RdRP, RNA‐dependent RNA polymerase.

Next, we multiplexed all three targets in a single reaction and further reduced the RNase P primers/probe concentrations by 50%. The limit of detection of this triplexed assay maintained the same sensitivity as the singleplex and duplexed versions, detecting 100% of replicates at 2 copies per reaction (Fig. 4A). This triplexed version (called rTEST COVID‐19 qPCR Allplex kit) also correctly identified all negative (30/30) and SARS‐CoV‐2 positive (30/30) samples during a clinical performance evaluation (Fig. 4B; Table S6).

### A rapid, RNA extraction‐free SARS‐CoV‐2 test from gargle samples

Although our triplexed SARS‐CoV‐2 test provides a streamlined workflow without sacrificing sensitivity, the test suffers from lengthy thermocycling conditions as well as bottlenecks in the workflow caused by the necessity to collect nasopharyngeal samples and extract viral RNA. To address these issues, we used our triplexed E, RdRP and human RNase P primer/probe sets in conjunction with a new master mix that enables fast thermocycling conditions and is optimized for crude RNA samples (One‐Step RT‐qPCR Direct Kit 2; SOLIS Biodyne). First, we developed an in‐house lysis buffer that does not utilize guanidine salts and therefore avoids downstream PCR inhibition and combined this with a short heat treatment (95°C for 7 min) to inactivate and lyse minimally invasive oral rinse/gargle samples that can be self‐collected. Next, we tested various sample inputs (3–8 μl) of inactivated gargle and found that 8 μl was the optimal gargle sample input volume (Fig. 4C). Lastly, we determined the optimal thermocycling conditions by comparing several different variations in time or temperature for reverse transcription (20, 15 or 10 min), denaturation (1 or 3 s) and annealing/extension (10 or 5 s; 60 or 62°C), which resulted in a rapid thermocycling protocol that takes less than 60 min (Fig. 4D).

With these optimized reaction conditions, we determined the LoD of this rapid, RNA extraction‐free RT‐qPCR assay by spiking gargle samples with a known concentration of heat‐inactived SARS‐CoV‐2 viral particles, processing the samples with our lysis buffer and heat inactivation, and adding 8 μl into a rapid RT‐qPCR reaction. The LoD test revealed our assay detected all samples containing 2 SARS‐CoV‐2 copies μl^−1^ gargle (or 16 copies per reaction); Fig. 4E). By comparing the clinical performance of the new test called rTEST COVID‐19 qPCR Rapid kit with an index test (rTEST Allplex) using a selected set of 105 SARS‐CoV‐2 positive and 94 SARS‐CoV‐2 negative gargle samples, we observed impressive concordance with rTEST Rapid correctly identifying all negative samples (94/94) and 98% of positive samples (103/105) Two samples containing low viral loads according to the index test (e.g. *C*_t_ = 34 and 38.6 for RdRP) were only detected by the rTEST Rapid E gene assay and were therefore classified as inconclusive (Fig. 4F; Table S7).

### Surveillance of primer and probe complementarity to SARS‐CoV‐2 genomes

As the amount of available SARS‐CoV‐2 sequences increased over time, we regularly checked the accuracy of our primer and probe sequences to verify the presence of potential sequence mutations that could have a detrimental impact on assay performance. One of our large scale *in silico* analysis of all GISAID sequences downloaded from 31.12.2019 to 14.10.2020 (133 243 sequences) revealed that > 99% of all SARS‐CoV‐2 sequences gave exact matches to our final primer/probe sets for E and RdRp genes (Table 2), suggesting near perfect complementarity to virtually all SARS‐CoV‐2 genomes prior to the emergence of the Alpha variant.

**Table 2:** Analysis of mismatches in primers/probes relative to SARS‐CoV‐2 genomes.

Variant(s) searched	Pre‐alpha variant	Delta variant	Omicron variant
Date range of search	31.12.2019–14.10.2020	01.11.2021–15.11.2021	23.11.2021–05.12.2021
**Primer name**	**Perfect match**	**Perfect match**	**Perfect match**
E_Sarbeco_F3	**Not searched**	35 617/35 807 99.5%	446/450 99.1%
E_Sarbeco_F7	132 704/133 243 99.6%	35 666/35 807 99.6%	450/450 100%
E_Sarbeco_R2	132 961/133 243 99.8%	35 682/35 807 99.7%	450/450 100%
E_Sarbeco_P1	132 778/133 243 99.7%	35 708/35 807 99.7%	450/450 100%
E_Sarbeco_P2	132 732/133 243 99.6%	35 703/35 807 99.7%	450/450 100%
RdRP_SARSr‐F2	132 162/133 243 99.2%	10/35 807 0.03%	450/450 100%
RdRP_SARSr‐R2	132 664/133 243 99.6%	18 455/35 807 51.5%	433/450 96.2%
RdRP_SARSr‐P2	132 819/133 243 99.7%	34 104/35 807 95.2%	450/450 100%
RdRP_SARSr‐P8	132 195/133 243 99.2%	34 181/35 807 95.5%	450/450 100%

Green boxes – primers with no mismatches or mismatches that have negligible impact on performance. Red boxes – primers with mismatches that may alter performance. Gray box – primer was not searched for sequence complementarity.

Since the emergence of new SARS‐CoV‐2 variants of interest and concern is frequently associated with mutations occurring in primer/probe binding sites, we actively monitored the mutations present in new variants. For example, we identified two mutations in the Delta variant that affected both RdRP primers: a T>A mutation in the fifth base from the 3′‐end of the reverse primer that was present in 48.5% of sequences and a G>A mutation in the penultimate base from the 3′‐end of the forward primer that was present in 99.7% of sequences (see results from our latest analysis on 35 703 Delta sequences spanning a 14‐day period from 01.11.2021 to 15.11.2021, Table 2). Internal testing revealed that the latter mutation did not cause the reaction to fail, but it did reduce the sensitivity of the assay (as demonstrated by a shift in *C*_t_ value compared with SARS‐CoV‐2 wild type), leading us to develop a Delta specific forward primer (see Table 1 and Table S1, RdRP_Delta‐F2) that resolved the reduced sensitivity. In regards to the Omicron variant that has rapidly displaced other variants, an analysis of 450 sequences deposited on GISAID on 05.12.2021 revealed near perfect complementarity with our E and RdRP primers and probes (Table 2). Overall, these analyses underscore not only the utility of these assays in detecting SARS‐CoV‐2 variants throughout the pandemic but also the necessity for PCR test developers to remain vigilant in surveying potential mutations in primer/probe binding sites and make adjustments accordingly.

### Differentiation of SARS‐CoV‐2 from influenza A and B

**Fig. 5:**
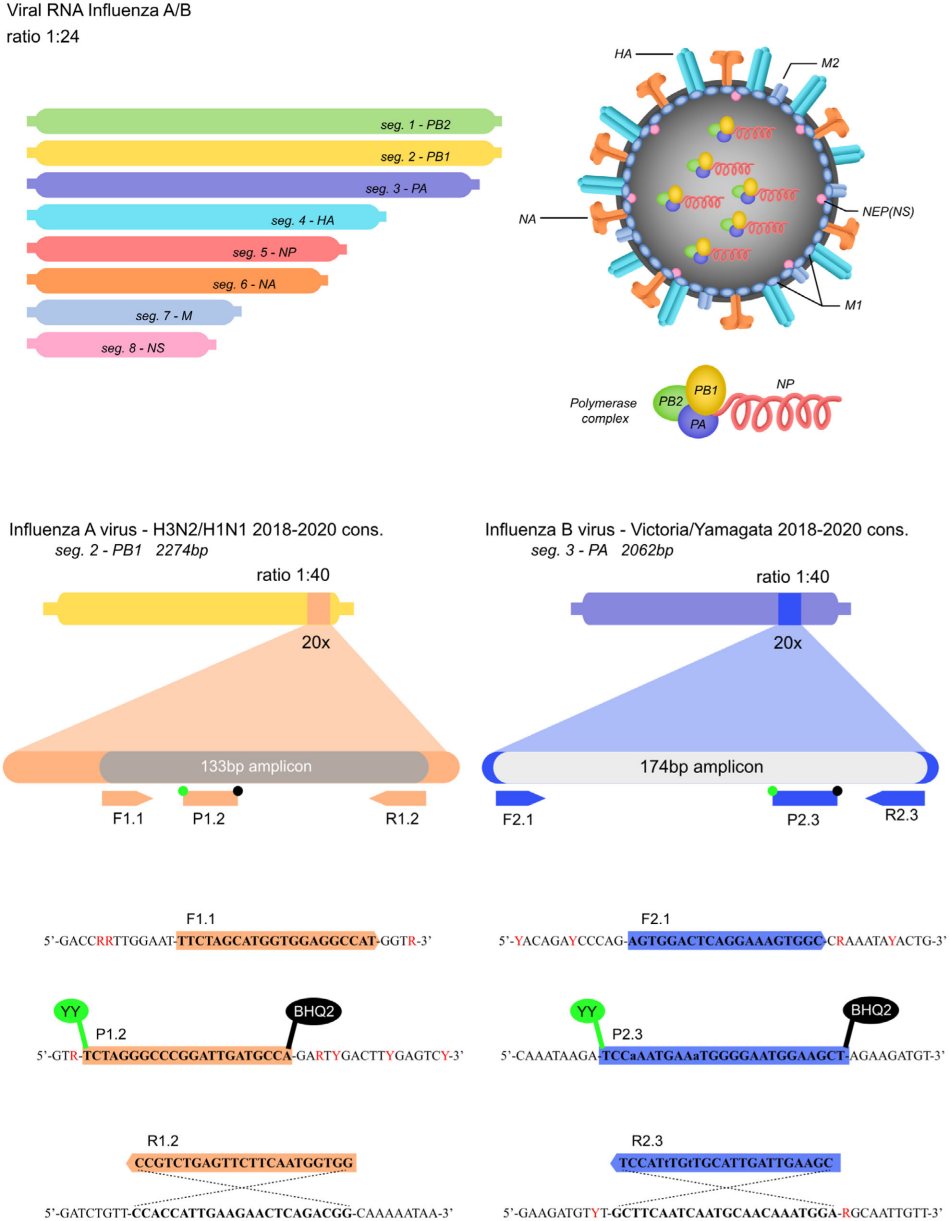
Schematic illustrating influenza A and B genome and regions targeted by RT‐qPCR primers and probes. (A) Schematic overview portrays the influenza A and B genome with PB1 and PA gene regions magnified to show the locations of primers and probes. Nucleotides labelled in red text indicate mixed bases in the consensus sequences for influenza A and B. BHQ2, black hole quencher 2; F, forward primer; HA, haemagglutinin; M, matrix protein; NA, neuraminidase; NP, nucleoprotein; NS, non‐structural protein; P, probe; PA, polymerase acidic protein; PB1, polymerase basic 1 protein; PB2, polymerase basic 2 protein; R, reverse primer; seg., segment; YY, Yakima Yellow^®^.

Since other respiratory pathogens such as seasonal influenza produce symptoms that overlap with SARS‐CoV‐2, it is important to have molecular diagnostics that can effectively differentiate between the two respiratory viruses. To develop an RT‐qPCR assay that could distinguish between SARS‐CoV‐2 and the influenza A and B, we conducted an extensive bioinformatic analysis of over 27 000 influenza A (H1N1 and H3N2) and over 8000 influenza B (Victoria and Yamagata) sequences of the PB1, PB2 and PA segments deposited in GISAID from 1.1.2018 to 24.6.2020. First, we identified the number of point mutations and missing bases for IAV (Figs S5 and S7) and IBV (Figs S6 and S8) and filtered out sequences containing a sum of point mutations and missing bases that was at least two standard deviations above the mean. Second, we shrunk the amount of sequences and reduced clonal bias by identifying geographical biases (Fig. S9). In these biased regions (i.e. regions that contained more than 100 sequences, log_10_ scale = 2), we identified sequence clones and used only one sequence per clade (cluster). After these two rounds of filtering to reduce biases introduced by gaps (i.e. mutations/missing bases) and sequence clones, we used a sliding window approach to find loci within gene segments of IAV (Fig. S10) and IBV (Fig. S11) that were conserved and long enough (at least 3 loci with 30 bp conserved stretches in a 200 bp segment) for suitable primer/probe design. With this approach, we determined that the PB1 segment for IAV and the PA segment for IBV contained several highly conserved areas with minimal amounts of mixed and unknown bases (Fig. 5), allowing us to develop RT‐qPCR primer/probe sets that capture the predominant seasonal influenza viruses while avoiding highly degenerate primer/probe sequences.

**Fig. 6:**
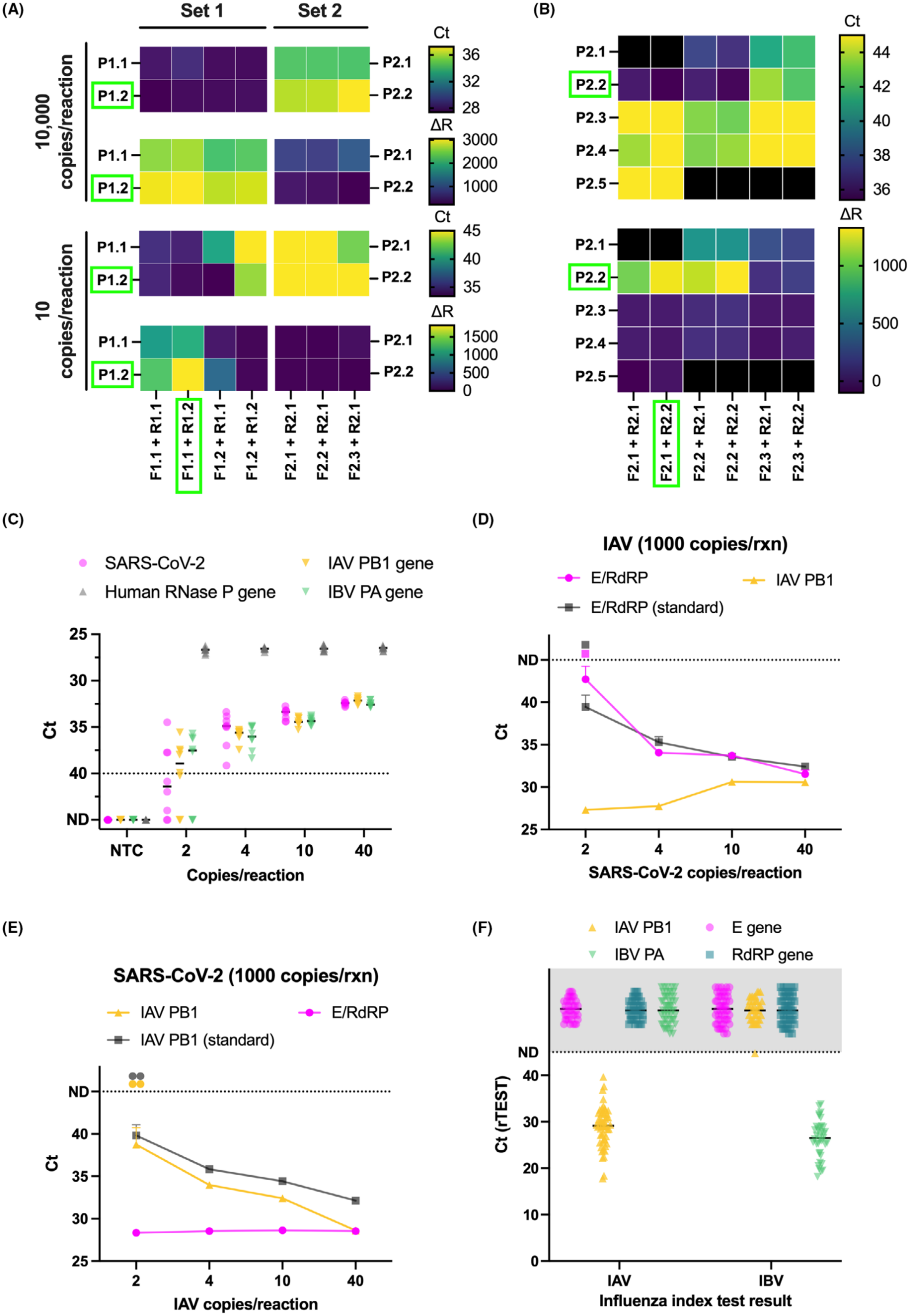
Optimization, analytical sensitivity and clinical performance of rTEST COVID‐19/FLU qPCR kit. A. Heatmaps illustrate combinatorial testing of two IAV primer/probe sets at either high or low viral input (10 000 versus 10 copies per reaction). B. Heatmaps show combinatorial testing of IBV primer/probe sets at low viral input (10 copies per reaction). In panels A, B, the best performing primer/probe combinations (highlighted by green rectangles) were selected based on *C*_t_ (darker colours denote higher sensitivity), fluorescent intensity (∆*R*, lighter colours correspond to higher intensity) and the number of replicates that amplified. C. Analytical sensitivity of the multiplexed SARS‐CoV‐2 E and RdRP (both labelled with FAM), IAV PB1 and IBV PA (both labelled with YY), and RNase P assay. The dotted line at *C*_t_ = 40 serves as a threshold after which amplification is considered invalid. D. Assessment of competitive interference of 1000 copies of IAV per reaction (500× LoD) on the analytical sensitivity of the SARS‐CoV‐2 E and RdRP assays multiplexed together. E. Assessment of competitive interference of 1000 copies of SARS‐CoV‐2 per reaction (500× LoD) on the analytical sensitivity of the IAV PB1 assay. F. Clinical performance of the rTEST COVID‐19/FLU qPCR kit. The dotted line and shaded area indicate samples that were not detected by a particular assay. *C*_t_, cycle threshold; E, envelope gene; IAV, influenza A; IBV, influenza B; PA, polymerase acidic protein; PB1, polymerase basic 1 protein; ND, not detected within 45 cycles; NTC, no template control; RdRP, RNA‐dependent RNA polymerase; Δ*R*, normalized fluorescent intensity.

Given the highly degenerate consensus sequences, we only identified two potential primer/probe sets for IAV (Sets 1 and 2 each containing several primer/probe combinations) that did not contain any degenerate bases. We screened these sets by using both high and low concentrations of template (10 000 versus 10 copies per reaction). Set 1 (F1.1, P1.2, R1.2) performed the best with lower *C*_t_ values and higher fluorescent intensities especially at low viral copy number (Fig. 6A). Introducing LNA‐modified nucleotides into the forward primer of Set 2 (F2.1) to raise its low melting temperature (53.9°C) provided no additional benefit (Fig. 6A). For IBV, we screened several primer/probe combinations using only a low input of template (10 copies per reaction) and found that using LNA‐modified nucleotides to elevate and normalize melting temperatures was beneficial for the reaction (e.g. comparison of R2.1 versus R2.2; Fig. 6B). With the optimal primer/probe sets for IAV and IBV, we tested incorporating a second dual probe into the reaction, but this resulted in only a marginal benefit for signal intensity and no change in sensitivity (Fig. S12A and B).

After finalizing the sets of primers and probes, we multiplexed the optimal primer/probes sets for IAV and IBV with both SARS‐CoV‐2 E and RdRP genes (both labelled with FAM) and determined the analytical sensitivity. We tested two multiplexed formats: (i) the first format allows the differentiation of SARS‐CoV‐2, IAV and IBV and consists of two multiplexed assays containing either SARS‐CoV‐2 E gene multiplexed with IAV PB1 gene and RNase P or SARS‐CoV‐2 RdRP gene multiplexed with IBV PA gene and RNase P; and (ii) the second format allows for the differentiation of SARS‐CoV‐2 and influenza (but not the distinction between IAV and IBV). This format consists of a single multiplexed reaction containing both SARS‐CoV‐2 E and RdRP genes (both labelled with FAM), IAV PB1 and IBV PA genes (both labelled with YY), and RNase P (Cy5). The first format with two multiplexed reactions yielded exceptional sensitivity with all multiplexed targets detecting every replicate at only 2 copies per reaction (Fig. S12C and D); while the second format with one multiplexed reaction had a slightly higher limit of detection for all targets of 4 copies per reaction (Fig. 6C).

Given reports of individuals being coinfected with both SARS‐CoV‐2 and influenza (Azekawa *et al*., 2020; Cuadrado‐Payán *et al*., 2020; Kim *et al*., 2020; Zheng *et al*., 2020; Wu *et al*., 2020), we sought to determine whether a high viral load of one virus would potentially interfere with the limit of detection of the other. For this competitive interference experiment, we conducted additional LoD experiments for both SARS‐CoV‐2 (both E and RdRP genes multiplexed together) and IAV in the presence of a high viral load (500× LoD) of the competing virus. High viral loads of the competing virus did not reduce detection sensitivity of either SARS‐CoV‐2 (Fig. 6D) or IAV (Fig. 6E) assays. We also tested the scenario of coinfection with high viral loads of each virus and whether this would lead to assay inhibition due to consumption of reagents (e.g. dNTPs, enzyme). In this experiment, we ran the multiplexed SARS‐CoV‐2 E, IAV and RNase P assay in parallel with the multiplexed SARS‐CoV‐2 RdRP, IBV and RNase P assay and conducted an LoD experiment starting with high viral loads (10 240 copies per rxn, 5000× LoD) and subsequent 4‐fold dilutions. All assays successfully detected their viral targets at all dilutions, including the most concentrated at 5000× LoD (Fig. S12E and F). At 5000× LoD, the fluorescent signal was noticeably diminished for all targets in the multiplexed assay indicating minor inhibition due to reagent consumption (Fig. S12G and H); however, this occurred late in the exponential phase of amplification so it did not impact the cycle threshold and thus the ability to detect the target.

We assessed the clinical performance of this test, called rTEST COVID‐19/FLU qPCR kit, on a selected set of 52 and 37 clinical samples of patients diagnosed with IAV and IBV, respectively, by a reference method used for routine testing by regional public health authorities of the Slovak Republic. Both the IAV PB1 and IBV PA gene assays correctly identified all positive samples (IAV PB1 = 52/52; IBV PA = 37/37; Fig. 6F; Table S8). One sample confirmed as IBV showed late amplification (*C*_t_ = 44.77) with the IAV assay; however, this would be deemed as negative in clinical practice. Importantly, there was no cross reactivity between SARS‐CoV‐2 and either IAV or IBV samples demonstrating that the test accurately differentiates between all three viruses. These assays displayed no cross‐reactivity to other coronaviruses and respiratory pathogens during wet‐lab cross‐reactivity experiments further highlighting their specificity (Table S3).

## Discussion

In this paper, we improved the original Charité SARS‐CoV‐2 RT‐qPCR protocol by correcting the mismatched bases, normalizing primer melting temperatures using LNA‐modified nucleotides and incorporating a human RNase P internal control to assess RNA extraction, RNA integrity and assay performance. Our revamped SARS‐CoV‐2 assays also contain technological novelties such as dual probes to enhance specificity and sensitivity, a room‐temperature stable master mix, and primer limited multiplexed assays that enable higher throughput testing while maintaining exceptional sensitivity. Moreover, we optimized a direct (RNA‐extraction‐free) version of this multiplexed assay that retains high sensitivity and accepts minimally invasive gargle samples, allowing self‐collection of samples and results in about an hour. To aid in differentiating SARS‐CoV‐2 from other respiratory pathogens that have overlapping symptomatology, we multiplexed our SARS‐CoV‐2 assays with primer/probe sets targeting the most common seasonal influenzas. These assays, outlined in Table 3, can provide labs with specific, ultrasensitive RT‐qPCR tests to scale their efforts to detect SARS‐CoV‐2 and influenza.

**Table 3:** Comparison between Charité protocol, vDetect and rTEST kits.

Kit/protocol	RNA extraction	Multiplexed	Dual probes	RNase P	RT stable	LoD (copies/reaction)^a^				Number of reactions for result	IVD CE
						E	RdRP	IAV/PB1	IBV/PA		
Charité	Yes	No	No	No	No	3.9^a^	3.6^a^	—	—	2	None
vDetect v1	Yes	No	No	No	No	8	8	—	—	2	05/2020
vDetect v2	Yes	No	No	Yes	No	2	2	—	—	2–3^d^	07/2020
*rTEST*											
Singleplex	Yes	No	Yes	Yes	Yes	2	2	—	—	2–3^d^	08/2020
Multiplex	Yes	Yes	Yes	Yes	Yes	2	2	—	—	1–2^e^	09/2020
Allplex	Yes	Yes	Yes	Yes	Yes	2	2	—	—	1	01/2021
Rapid	No	Yes	Yes	Yes	Yes	2^b^	2^b^	—	—	1	11/2021
COVID‐19/Flu	Yes	Yes	Yes	Yes	Yes	2–4^c^	2–4^c^	2–4^c^	2–4^c^	1–2^e^	10/2020

^a^ The LoDs reported for Charité are theoretical 95%LoDs derived from a probit curve; the LoDs reported for vDetect and rTEST kits are 100%LoDs derived from empirical wet lab tests.

^b^ LoD expressed as copies μl^−1^ gargle input (equivalent to 16 copies per reaction).

^c^ LoD dependent on testing format.

^d^ Number of reactions dependent on necessity of RNase P.

^e^ Number of reactions dependent on testing format.

For each category, the attributes for each kit are ranked using a colour scale: green (excellent or positive attribute), yellow (good), orange (fair), and red (poor or negative attribute).

### Redesigned and revamped RdRP and E gene primer/probe assays

Being the first RT‐qPCR test for SARS‐CoV‐2 to be published (Corman *et al*., 2020) and approved by the WHO (World Health Organization (WHO)), the Charité protocol was developed without access to SARS‐CoV‐2 isolates or clinical specimens as well as a paucity of genomic sequences; therefore, the assay design relied on genetic sequences from closely related SARS‐CoV and bat‐related coronaviruses, which resulted in the placement of several degenerate bases in the RdRP primers and probe. Similar to other reports (Pillonel *et al*., 2020; Vogels *et al*., 2020), our bioinformatic analysis of SARS‐CoV‐2 genomes revealed these degenerate bases in the RdRP forward and reverse primers resulted in mismatched bases, potentially contributing to the reduced sensitivity of the RdRP assay that others have observed (Etievant *et al*., 2020; Jung *et al*., 2020; Nalla *et al*., 2020; Pillonel *et al*., 2020; Vogels *et al*., 2020). Another problem with the Charité RdRP assay stems from the low melting temperature of the reverse primer. This difference in melting temperatures between the forward and reverse primers (8.5°C Tm difference) can result in altered patterns of annealing and consequently reduced efficiency and sensitivity. This deficiency in primer design, as pointed out by the authors of the Charité protocol (Corman and Drosten, 2020), is the more likely culprit responsible for the reduced sensitivity of the RdRP assay, since PCR is generally tolerant of mismatches that occur in the middle and 5′‐end of primers (as is the case here). A recent report that analyses the Charité RdRP assay in detail, purports that the underlying cause of the reduced sensitivity is primarily the mismatched base in the RdRP reverse primer (i.e. S, which is defined as G/C, but is a T in the SARS‐CoV‐2 consensus sequence). Whereas the two mismatches in the RdRP probe or the lower melting temperature of the RdRP reverse primer (i.e. Tm mismatch) did not influence the performance of the assay (Bustin *et al*., 2021). Although we did not conduct experiments to determine the root cause of the suboptimal RdRP assay, we found that correcting the mismatched bases in both forward and reverse primers and redesigning the reverse primer to ensure a higher Tm remedied the performance issues with this assay and resulted in comparable performance to the E gene assay.

Due to reports of commercially supplied primers/probes being contaminated with synthetic positive controls for the E gene (Fischer *et al*., 2020; Huggett *et al*., 2020; Mögling *et al*., 2020; Wernike *et al*., 2021), we also redesigned the forward primer of the E gene so that it would not amplify the most common synthetic positive controls. This presented challenges because the small size of the E gene and AT‐rich nucleotide content provides few choices for designing full length primers with optimal annealing temperatures. To circumvent these design limitations, we incorporated LNA‐modified thymine bases into the 5′‐end of the forward primer, which allowed us to shorten the length of the primer to eliminate any overlap with the Charité E gene forward primer (and consequently E gene synthetic positive controls), while still maintaining the optimal duplex annealing temperature. This new E gene forward primer design offers an innovative solution to eliminate potential issues related to contamination with synthetic positive control without having to develop an assay that targets a different gene. The use of LNA‐modified nucleotides builds on our previous work using LNA bases to enhance mismatch discrimination in distinguishing the B.1.1.7 (alpha) variant from wild‐type SARS‐CoV‐2 (Boršová *et al*., 2021) and highlights the versatility of LNAs to normalize primer melting temperatures and provide flexibility in primer design for problematic targets.

### Dual probes increase sensitivity and specificity of SARS‐CoV‐2 RT‐qPCR assays

Prior research suggests that introducing a second TaqMan hydrolysis probe into the RT‐qPCR reaction, labelled with the same reporter dye and placed either in tandem or on the opposite strand as the first probe, results in an additive increase in fluorescent intensity (Nagy *et al*., 2017) and can even enhance the sensitivity of the assay (Yip *et al*., 2005). Consistent with these reports, we observed that a dual probe when hybridized in tandem with the original probe roughly doubled the fluorescent intensity and increased sensitivity by reducing the average *C*_t_ value at a given copy number per reaction. However, in our hands, a second hydrolysis probe placed antisense to the first probe was detrimental to sensitivity, although this effect could be specific to our assay. Interestingly, the dual probes used in our RdRP assay overlap, yet still provide additive gains in fluorescent intensity and enhanced sensitivity. The novel finding that overlapping dual probes provide similar benefits to dual probes that hybridize in tandem, affords users with additional flexibility to design dual probe assays, especially when targeting difficult templates such as short templates and those containing mutations (e.g. viruses) or suboptimal nucleotide content (e.g. AT rich, low complexity sequences).

A pertinent benefit of using dual probes is the inherent increase in specificity of the assay. This is particularly important when developing RT‐qPCR assays for detection of viruses that have a natural propensity to mutate. Mutations in the viral genome that result in mismatches in primer‐ or probe‐binding regions can be detrimental to the performance of an assay and are part of the rationale for public health bodies to recommend multi‐gene target assays for detection of SARS‐CoV‐2. Indeed, there is a plethora of evidence showing that SARS‐CoV‐2 mutations can severely affect the performance of RT‐qPCR assays (Artesi *et al*., 2020; Jaroszewski *et al*., 2020; Khan and Cheung, 2020; Wang *et al*., 2020b), and that the accumulation of mutations over time and geographical location can exacerbate this problem (Nayar *et al*., 2021). One example of this phenomena involves the emergence of the B.1.1.7 (alpha) lineage first discovered in the United Kingdom. This variant was first identified because it contains a deletion in the spike gene that caused a rising number of RT‐qPCR assays to fail – so‐called spike gene target failures (Bal *et al*., 2021; Boršová *et al*., 2021; Brejová *et al*., 2021). By utilizing an additional dual hydrolysis probe, our SARS‐CoV‐2 assays contain an additional layer of specificity such that any potential mutation that results in a mismatch in one probe binding region is compensated by the other probe.

### Rapid, RNA extraction‐free detection of SARS‐CoV‐2 from gargle samples

Most RT‐qPCR tests for SARS‐CoV‐2 detection are limited by the requirement to extract viral RNA from the sample, a procedure that is laborious, time‐consuming, costly and susceptible to bottlenecks from reagent/kit shortages and supply constraints. Our rapid rTEST utilizes a viral inactivation step comprising an optimized lysis buffer and heat followed by a rapid thermocycling program that provides users with results in about an hour of sample collection. By optimizing the sample input volume and thermocycling parameters, we maintained excellent sensitivity relative to the same RT‐qPCR assay conducted after RNA extraction. Moreover, this assay is compatible with gargle samples that can be self‐collected by individuals in a minimally‐invasive manner, thus circumventing the need for sample collection teams that are exposed to potentially infectious people. We also verified that rTEST Rapid is compatible and displays similar performance with alternative sample input media, including H2O, a sample preservation solution containing Hanks' Balanced Salt Solution (Mole Biosciences, Hangzhou, China), and a VTM containing PBS buffer (pH = 7.3), antibiotics and foetal bovine serum (COROTEST, Labmediaservis, Jaroměř, Czech Republic), illustrating that rTEST Rapid can easily be integrated into existing sample collection workflows. This approach could supplement the conventional testing workflow of RNA extraction followed by RT‐qPCR and enable rapid screening of patients and frequent surveillance of larger populations in a scalable manner.

### Differentiating SARS‐CoV‐2 from influenza A and B

The circulation of other respiratory pathogens provides a challenging scenario for physicians in correctly distinguishing individuals infected with SARS‐CoV‐2 from those infected with other pathogens such as influenza because they often have overlapping symptomatology. This problem is exaggerated by reports of people being co‐infected with both SARS‐CoV‐2 and influenza (Azekawa *et al*., 2020; Cuadrado‐Payán *et al*., 2020; Kim *et al*., 2020; Zheng *et al*., 2020; Wu *et al*., 2020), suggesting that testing positive for another respiratory pathogen does not preclude the absence of a SARS‐CoV‐2 infection. Therefore, there is a need for diagnostic tools to accurately differentiate SARS‐CoV‐2 from other respiratory pathogens especially seasonal pathogens like influenza. To address this challenge, we conducted an extensive bioinformatic analysis of over 35 000 influenza A and B sequences emphasizing sequences arising in the past two years to ensure high representation of recent cases. This analysis identified highly conserved targets in the PB1 (IAV) and PB (IBV) segments that are ideal targets for RT‐qPCR primers and probes. We also multiplexed these assays with our SARS‐CoV‐2 E and RdRP assays to create two reaction formats that provide unique benefits depending on the required throughput and necessity to distinguish between IAV and IBV. Since individuals can be co‐infected by both SARS‐CoV‐2 and influenza (Azekawa *et al*., 2020; Cuadrado‐Payán *et al*., 2020; Kim *et al*., 2020; Zheng *et al*., 2020; Wu *et al*., 2020), it is important to highlight that these assays are robust against competitive interference from high viral loads of any one virus, thus allowing co‐detection of both viral targets in a single sample regardless of relative viral loads.

## Conclusion

Early in the SARS‐CoV‐2 pandemic, several RT‐qPCR protocols were published by reference laboratories and public health bodies, enabling countries to quickly setup diagnostic workflows necessary to identify the novel coronavirus. While these protocols provided unquestionable benefits and formed the basis for many commercial RT‐qPCR tests, several issues emerged regarding sensitivity and specificity. This paper outlined the development of a series of RT‐qPCR tests based on the protocol developed by the Charité Institute of Virology. We remedied some of the deficiencies of this original assay by correcting mismatches in the primers, using LNA‐modified bases to normalize suboptimal annealing temperatures and incorporating dual probe technology to boost fluorescence signal and sensitivity, while also offering an extra layer of protection against mismatches produced by mutations in probe‐binding regions. Our multiplexed assays, which also contain an RNase P internal control, drastically reduce hands‐on‐time and conserve laboratory resources without sacrificing sensitivity. Some of the tests contain a room temperature‐stable master mix with lyophilized primers/probes stabilized with decoy nucleic acids placing them among only a few RT‐qPCR tests that do not require cold chain shipping and storage. These assays can be utilized in a direct, rapid testing workflow using minimally invasive, self‐collected gargle samples with little impact on sensitivity. Moreover, we multiplexed these SARS‐CoV‐2 assays with influenza A and B assays to facilitate rapid differentiation of these respiratory pathogens that pose challenges for healthcare practitioners to identify. These novel, room temperature‐stable RT‐qPCR tests can provide users with a powerful tool to detect SARS‐CoV‐2 rapidly and accurately in the next phase of the pandemic.

## Experimental procedures

### Bioinformatic analysis and primer design

Initially, we downloaded 505 SARS‐CoV‐2 sequences from the GISAID repository (accessed on 14 March 2020) and aligned them to the Wuhan reference sequence (NCBI ID: NC_045512.2) using the MAFFT alignment tool (with the parameter – auto)(Nakamura *et al*., 2018). We called the 95% consensus sequences using SeaView (Gouy *et al*., 2010) and compared with the primer/probe sequence in the original Charité protocol. Degenerate bases in the original Charité protocol were replaced with bases complementary to the consensus sequence (Fig. 1). As the amount of available SARS‐CoV‐2 sequences increased over time, we regularly checked the accuracy of our primer and probe sequences to verify the presence of potential sequence mutations that could have a detrimental impact on assay performance.

To design primers and probes specific to IAV and IBV, we downloaded 11 889 H1N1 and 15 498 H3N2 sequences (IAV) as well as 4610 Victoria and 3547 Yamagata sequences (IBV) of the PB1, PB2 and PA segments. All sequences were deposited in GISAID from 1.1.2018 to 24.6.2020 ensuring the primers/probes would target the most recent circulating influenza strains. We focused on sequences deposited in the past three years to ensure adequate coverage of the minimum time necessary for new antigenic variants to evolve (Petrova and Russell, 2018). For our analysis, we only used sequences derived from human bodily fluids (e.g. sputum, nasal mucus), since sequences from egg‐grown vaccines are susceptible to mutations (Petrova and Russell, 2018). Next, we wrote custom bash scripts to align these GISAID sequences to reference influenza strains using the MAFFT alignment tool (v7.453, released 2019/Nov/8, with the parameter – auto) (Nakamura *et al*., 2018) and then to verify the quality of the sequences and filter outliers. The following reference sequences were used: A/Guangdong‐Maonan/SWL1536/2019 (H1N1; reference seq: EPI_ISL_390379), A/Hong Kong/2671/2019 (H3N2; reference seq: EPI_ISL_391201), B/Washington/02/2019 (B/Victoria lineage; reference seq: EPI_ISL_362540) and B/Kyiv/9/2018 (B/Yamagata lineage; reference seq: EPI_ISL_296613). With the exception of the reference sequence for B/Yamagata lineage, these reference sequences were selected due to their recommendation for tri‐/quadra‐valent influenza vaccine development for use in the 2020–2021 northern hemisphere influenza season. Since the last sequence used for B/Yamagata vaccine development (e.g. B/Phuket/3073/2013) is outside our time period and only has the haemagglutinin (HA) segment sequenced, we instead used B/Kyiv/9/2018 as the reference sequence.

Next, to reduce errors arising from sequences containing many point mutations and unknown bases, we filtered out sequences that contained more than two standard deviations above the mean number of point mutations and unknown bases. To explore potential geographic sampling biases, we also used the phylogenetics RAxML tool (version 8.2.12) (Stamatakis, 2014) to identify enriched clones and subsequently reduced clonal bias by using only one clone per clade (cluster). Lastly, we computed the degree of degeneracy (i.e. the proportion of nucleotides different than the reference) and averaged those numbers using a sliding window approach (30 bp window with a shift by 10 bp) to find loci within segments that displayed long enough conservation for primer design (e.g. at least 3 × 30 bp conserved regions in a 200 bp segment). Details of the analysis and scripts are located on Github (https://github.com/MultiplexDX/flu‐mafft‐check‐quality). This analysis enabled us to identify highly conserved regions while avoiding regions containing degenerate bases and high rates of mutation for primer/probe design.

For all primers and probes, we checked the melting temperatures (*T*_m_), GC content, propensity to form homo‐/hetero‐dimers and stable secondary structures and hairpins using the IDT OligoAnalyzer™ tool (https://www.idtdna.com/pages/tools/oligoanalyzer) and the mFold server (Zuker, 2003) (http://www.bioinfo.rpi.edu/applications/mfold/). We also incorporated LNA‐modified nucleotides into select primers and probes to raise Tm, normalize Tm across different primer pairs and stabilize binding dynamics. Primers and probes were synthesized at MultiplexDX, s.r.o. (Bratislava, Slovakia; https://www.multiplexdx.com/).

### Surveillance and Inclusivity analysis of primer and probe complementarity to SARS‐CoV‐2 genomes

We engaged in routine surveillance of the primer/probe‐binding sites in SARS‐CoV‐2 sequences to ensure sequence complementarity and performance of our assays. This included regular tracking of emerging SARS‐CoV‐2 variants and whether their lineage‐defining mutations resulted in primer/probe mismatches, with a particular emphasis on variants located in geographical locations where our tests were in use. Our primary sources for routine monitoring were Nextstrain.org (Hadfield *et al*., 2018), CoVariants.org (Hodcroft, 2021) and Outbreak.info (Mullen *et al*., 2020), which we used to track prevalence and geographical location, lineage defining synonymous and non‐synonymous mutations, and the percentage of sequences containing particular mutations. Moreover, we periodically conducted more thorough *in silico* inclusivity analyses by downloading SARS‐CoV‐2 genome sequences from GISAID, aligning them to the Wuhan reference sequence (NCBI ID: NC_045512.2) using the MAFFT alignment tool, and then using custom R scripts to verify primer/probe‐binding site complementarity. Instructions and scripts are posted on Github (https://github.com/MultiplexDX/corona_cheks).

### Sample processing, RT‐qPCR reaction setup and thermocycling

#### vDetect v1

RT‐qPCR reactions were optimized on a CFX96 (Bio‐Rad), QuantStudio 5 (Agilent Technologies, CA, USA) and Mx3005P (Agilent Technologies) real time PCR detection systems using the 1Step RT qPCR Probe ROX L Kit (Cat. No. QOP0201, highQu, Germany). For E and RdRP genes, the reaction mixture was prepared according to the manufacturer´s recommendations comprised of 10 µl of 2× HighQu Master Mix, 2 µl of RT3 Mix, 2 µl of primers/probe mix, 1 µl of PCR water and 5 µl of sample in a 20 µl total volume. One‐step RT‐qPCR assays were conducted with the following cycling conditions: 50°C for 10 min for reverse transcription, 95°C for 3 min and 45 cycles of 95°C for 5 s and 60°C for 20 s. The sequences of primers and probes are shown in Table 1 and Table S1.

#### vDetect v2

RT‐qPCR reactions were optimized on a CFX96 (Bio‐Rad), QuantStudio 5 (Agilent Technologies), Mx3005P (Agilent Technologies) and AriaMx (Agilent Technologies) real time PCR detection systems using Brilliant III Ultra‐Fast QRT‐PCR Master Mix (Cat. No. 600884; Agilent Technologies). For E, RdRP and RNase P genes, the reaction mixture was prepared according to the manufacturer´s recommendations comprised of 10 µl of 2× Brilliant III Ultra‐Fast QRT‐PCR Master Mix, 0.3 µl of 2 µM ROX, 0.2 µl of 100 mM DTT, 1 µl of RT/RNase Block, 2 µl of primers/probe mix, 1.5 µl of PCR water and 5 µl of sample in a 20 µl total volume. One‐step RT‐qPCR assays were conducted with the following cycling conditions: 50°C for 30 min for reverse transcription, 95°C for 3 min and 45 cycles of 95°C for 5 s and 60°C for 20 s. The sequences of primers and probes are shown in Table 1 and Table S1.

#### rTEST singleplex, multiplex and allplex

RT‐qPCR reactions were optimized on a Mx3005P (Agilent Technologies), QuantStudio 5 (Agilent Technologies) and AriaMx (Agilent Technologies) real‐time PCR detection systems using SOLIScript^®^ 1‐step CoV Kit (Cat. No. 08‐65‐00250; SOLIS BioDyne, Tartu, Estonia). For all genes, the reaction mixture was prepared according to the manufacturer´s recommendations comprised of 4 µl of 5× One‐step Probe CoV Mix (ROX), 0.5 µl of 40× One‐step SOLIScript^®^ CoV Mix, 2 µl of primers/probe mix, 8.5 µl of PCR water and 5 µl of sample in a 20 µl total volume. One‐step RT‐qPCR assays were conducted with the following cycling conditions: 55°C for 10 min for reverse transcription, 95°C for 10 min and 45 cycles of 95°C for 15 s and 60°C for 30 s. The sequences of primers and probes are shown in Table 1 and Table S1.

#### rTEST rapid

Prior to running the RNA‐extraction‐free RT‐qPCR reactions, individuals known to be negative for SARS‐CoV‐2 self‐collected mouth rinse/gargle specimens by gargling 5 ml of isotonic saline solution (0.9% w/v in sterile H_2_O) for 60 s using instructions in a previously described protocol with minor modifications (Goldfarb *et al*., 2021). These gargle specimens were then spiked with heat‐inactivated SARS‐CoV‐2 viral particles (see positive controls below) and mixed with an in‐house 10× Rapid Lysis Buffer (100 mM of Tris–HCl, pH = 8.8; 100 mM of Na_2_EDTA, pH = 8.0; 4.5 mg ml^−1^ of Pronase in H_2_O; 100 µg ml^−1^ of Yeast mixed RNA in 100 mM of Na citrate; 5 mM of TCEP‐HCl; final pH of lysis buffer adjusted to pH = 7.0 with NaOH) in a 9:1 ratio (i.e. 90 μl of gargle:10 μl of lysis buffer) and incubated at room temperature for 3 min. Samples were subsequently heat treated at 95°C for 7 min to inactivate the viral particles and to release viral RNA into solution. Post‐heating, the samples were allowed to cool for a few seconds, centrifuged for 1 min and the appropriate sample volume (e.g. 8 μl) of the supernatant was aspirated and directly added to the RT‐qPCR reaction.

RT‐qPCR reactions for the rTEST Rapid kit were optimized on a QuantStudio 5 (Agilent Technologies), and AriaMx (Agilent Technologies) real time PCR detection systems using the One‐step RT‐qPCR Direct Kit 2 (Cat. No. 08‐78‐00250; SOLIS BioDyne). For all genes, the reaction mixture was prepared according to the manufacturer´s recommendations comprised of 4 µl of 5× One‐step Probe Direct Mix 3 (ROX), 0.5 µl of 40X One‐step RT Direct Mix 2 and 2 µl of primers/probe mix. We optimized the volume of gargle sample input by testing 2, 3, 4, 5, 6, 7 and 8 µl of inactivated gargle sample and adjusting the total reaction volume to 20 µl by adding the corresponding volume of PCR water. One‐step RT‐qPCR assays were conducted with the following cycling conditions: 50°C for 15 min for reverse transcription, 95°C for 10 min and 45 cycles of 95°C for 1 s and 60°C for 5 s. The sequences of primers and probes are shown in Table 1 and Table S1.

### One‐step RT‐qPCR optimization

The optimal RT‐qPCR conditions described above are the results of optimizing the thermal profile and composition of the reaction mixture. Optimal RT‐qPCR conditions were determined for each kit separately and the individual optimization steps are described in Table S2. Not all alternative thermal profiles were tested in combination with each additive/alteration. In the process of the thermal profile optimization, the composition of the reaction mixture recommended by the manufacturer was used. Additives or alterations in reaction mixture composition were tested using an optimized thermal profile (marked with bold).

### SARS‐CoV‐2 and influenza positive and negative controls

The SARS‐CoV‐2 positive control material developed by the Joint Research Centre (Cat. No. EURM‐019; JRC) was used in early development of the vDetect v1. assays. The JRC positive control contains single stranded RNA fragments (approximately 6.0 × 10^7^ copies µl^−1^) that can be amplified by several of the early WHO‐recommended RT‐qPCR protocol (including the E and RdRP assays from developed by the Charité).

The EDX SARS‐CoV‐2 Standard (Cat. No. COV019; Exact Diagnostics, Fort Worth, TX, USA) was used as a positive control for test optimization and LoD experiments. The EDX SARS‐CoV‐2 Standard contains synthetic RNA transcripts of five gene targets (E, N, ORF1ab, RdRP and S Genes) in concentrations of 200 copies µl^−1^. The synthetic RNA transcripts are in a matrix of genomic DNA allowing validation of the entire assay workflow including extraction, amplification and detection. The EDX SARS‐CoV‐2 Negative (Cat. No. COV000; Exact Diagnostics) reference material containing a synthetic matrix and genomic DNA at a concentration of 75 copies/µl was used to dilute the positive control materials to the desired concentrations.

‘AMPLIRUN^®^ INFLUENZA A H3 RNA CONTROL’ (Vircell Microbiologists, Granada, Spain) containing the complete IAV genome, diluted to 200 copies µl^−1^, was used as a control template for IAV assay optimization and LoD experiments. Viral RNA isolated from a MDCK cell line infected with Influenza B 17/381 was diluted to 200 copies/µl and used as template for IBV detection. Isolation of Influenza B 17/381 was performed with the QIAamp Viral RNA Mini Kit (Qiagen, Hilden, Germany) according to the manufacturer´s recommendations.

The PC BMC5 positive control consists of lyophilized isolated full genomic RNA of SARS‐CoV‐2 virus strain Slovakia/SK‐BMC5/2020 (available at https://www.european‐virus‐archive.com/virus/sars‐cov‐2‐strain‐slovakiask‐bmc52020) spiked with human RNA extracted from the human cell line A549. To determine the minimum stability of the lyophilized positive control and thus of the diagnostic kit at room temperature, three versions of the PC BMC5 were prepared: pure positive control, positive control stabilized by addition of baker´s yeast tRNA in a final concentration of 20 µg ml^−1^ and positive control stabilized by addition of salmon sperm DNA in a final concentration of 100 µg ml^−1^. After lyophilization, the stability of the positive control stored at room temperature for 0, 1, 4, 7, 21 and 33 days was tested by RT‐qPCR and was compared with a fresh, non‐lyophilized positive control.

The PC4.01 positive control consists of lyophilized isolated full genomic RNA of SARS‐CoV‐2, IAV, IBV, spiked human RNA extracted from the human cell line A549 and a stabilizer (baker´s yeast tRNA or salmon sperm DNA). The full genomic RNA of IAV and IBV was isolated from MDCK cell lines infected with IAV and IBV obtained from the National Influenza Centre, National Public Health Authority of Slovak Republic in Bratislava (Bratislava, Slovakia). Viral RNA was isolated using the QIAamp Viral RNA Mini Kit (Qiagen). The PC BMC5 and PC4.01 positive controls were diluted to concentrations yielding *C*_t_ values in the range of 28–35.

For rTEST Rapid test optimization and LoD experiments, NATtrol™ SARS‐Related Coronavirus 2 (SARS‐CoV‐2) Stock (Cat. No. NATSARS(COV2)‐ST; ZeptoMetrix, New York, USA) was used as a positive control. The stock is composed of a proprietary matrix with purified, intact viral particles (SARS‐CoV‐2 isolate: USA‐WA1/2020; target concentration of 1000 copies µl^−1^) that have been chemically modified, rendering them non‐infectious and refrigerator stable.

### Analytical sensitivity (limit of detection)

Evaluation of analytical sensitivity (limit of detection) was performed using 8 replicates over multiple concentrations, and 24 additional replicates were performed at concentrations spanning the level with 95% detection. In the case of vDetect v1., the dilutions were prepared by serial dilutions of the JRC positive control stock standard using matrix from SARS‐CoV‐2 negative samples, resulting in samples with concentrations of 40 copies μl^−1^ (= 200 copies per reaction), 8 copies μl^−1^ (= 40 copies per reaction), 1.6 copies μl^−1^ (= 8 copies per reaction) and 0.25 copies μl^−1^ (= 1.25 copies per reaction).

The dilutions of all the other kits, with the exception of rTEST Rapid, were prepared by serial dilutions of the stock standard, resulting in samples with concentrations of 8 copies μl^−1^ (= 40 copies per reaction), 2 copies μl^−1^ (= 10 copies per reaction), 0.8 copies μl^−1^ (= 4 copies per reaction) and 0.4 copies μl^−1^ (= 2 copies per reaction) that were used in the analytical sensitivity test. The EDX SARS‐CoV‐2 Negative reference material was used to dilute the positive control materials to the desired concentrations.

For rTEST Rapid, the NATtrol™ SARS‐CoV‐2 stock was diluted in SARS‐CoV‐2 negative gargle sample (90 μl) mixed with 10× Rapid Lysis Buffer (10 μl). A separate sample was prepared for each dilution with final concentrations of 40 copies μl^−1^ of gargle, 10 copies μl^−1^ of gargle, 4 copies μl^−1^ of gargle, 2 copies μl^−1^ of gargle and 1 copy per μl of gargle. After inactivation, 8 μl of inactivated gargle sample was used in the direct RT‐qPCR LoD test.

### Cross‐reactivity to other coronaviruses and respiratory viruses (test specificity)

Evaluation of specificity (potential cross‐reactivity to other coronaviruses and respiratory viruses) was performed using the Coronavirus RNA specificity panel (Cat. No. 011N‐03868; EVAg, European Virus Archive – Global), which contains RNA derived from cell culture from the following coronaviruses: HCoV‐Nl63, HCoV‐229E, HCoV‐OC43, MERS‐CoV, SARS‐CoV HKU39849 and SARS‐CoV‐2 each provided in a separate tube. A set of respiratory viruses (AmpliRun^®^ PCR controls; Vircell Microbiologists) containing purified complete RNA from SARS Coronavirus (Cat. No. MBC090), MERS Coronavirus (Cat. No. MBC132), Influenza A H1N1 (Cat. No. MBC028), Novel Influenza A H1N1 (Cat. No. MBC082), Influenza A H3N2 (Cat. No. MBC029), Influenza A H5N1 (Cat. No. MBC052), Influenza B (Cat. No. MBC030), Human parainfluenza 1 (Cat. No. MBC105), Respiratory syncytial virus subtype A (Cat. No. MBC041) and Rhinovirus (Cat. No. MBC091) were used to assess cross‐reactivity to respiratory viruses. Each pathogen was provided in a lyophilized format in a separate tube at a concentration range of 12 500–20 000 copies μl^−1^. Each specificity test contained at least one or more positive controls such as the EDX SARS‐CoV‐2 Standard, PC BMC 5 and/or JRC. For each of the indicated viruses, all assays were performed in at least triplicates, except for the PC BMC 1 and JRC positive controls, which were ran in duplicates.

### Competitive interference between SARS‐CoV‐2 and influenza assays

To determine if coinfection of both SARS‐CoV‐2 and influenza could lead to competitive interference between assays, we conducted LoDs for the SARS‐CoV‐2 assay (both E and RdRP combined) in the presence of a high viral load of influenza A (i.e. 1000 copies per reaction, 500× LoD) as well as the influenza A assay in the presence of a high viral load of SARS‐CoV‐2 (i.e. 1000 copies per reaction, 500× LoD). The mixed sample was prepared after RNA extraction and comprised of the positive controls described above by conducting serial dilutions with the diluent containing a fixed amount of high viral load sample to ensure 1000 copies per reaction in each diluted sample. To evaluate whether high viral loads of all viruses in a single sample would result in assay inhibition due to reagent depletion, we prepared a sample containing RNA from SARS‐CoV‐2, influenza A and influenza B at a high viral load of approximately 10 240 copies per reaction as determined by a PCR efficiency curve. We then took this sample and made four‐fold dilutions until reaching approximately 40 copies per reaction for each virus.

### Clinical evaluation

#### vDetect v.1

Evaluation of the clinical performance for the vDetect COVID‐19 RT‐qPCR kit was performed for both the E gene screening test and the RdRP gene confirmatory test in two independent laboratories using a selected set of 38 SARS‐CoV‐2 positive samples and 54 SARS‐CoV‐2 negative samples. Viral RNA was extracted from these samples using the Quick‐RNA™ Viral 96 Kit (Cat. No. R1040; Zymo Research, Irvine, CA, USA). The results of the vDetect v.1 test were benchmarked against an index test (E and RdRP gene targets from the original Charité protocol) and utilizing the GoTaq 1‐Step RT‐qPCR System (Promega, Madison, WI, USA). Validation of the vDetect COVID‐19 RT‐qPCR kit was performed independently in two separate laboratories using the same workflow described above: laboratory 1 (Biomedical Research Center – Slovak Academy of Sciences, B. Klempa) and laboratory 2 (Comenius University Science Park, T. Szemes).

#### rTEST

Clinical validation of the rTEST COVID‐19 qPCR kit was conducted using the same selected set of 38 SARS‐CoV‐2 positive samples and 54 SARS‐CoV‐2 negative samples. The samples were thawed to re‐extract RNA using the RNAdvance Viral Kit (Cat. No. C63510; Beckman Coulter, Brea, CA, USA) and the Biomek i5 Automated Workstation (Beckman Coulter) and then the same index test (vDetect v.1) was performed and compared with the original results using the same test to assess for RNA degradation. The new index test results served as the benchmark to assess performance of the rTEST.

The Laboratory for RNA Molecular Biology at Rockefeller University conducted an independent clinical evaluation of the rTEST COVID‐19 qPCR kit using a set of 15 SARS‐CoV‐2 positive and 15 SARS‐CoV‐2 negative nasopharyngeal swabs collected during routine testing at the New York City Department of Health and Mental Hygiene (NYC DOHMH). RNA was extracted using a bead‐based isolation protocol (SeraSil‐Mag, Cat. N. 29357369, Cytiva), and then all samples were ran in parallel to compare the rTEST COVID‐19 qPCR kit with the index test, US CDC 2019‐nCoV Kit (IDT, #10006713) using the TaqPath™ 1‐Step RT‐qPCR Master Mix (Thermo Fisher, #A15299) on a Stratagene Mx3000P instrument (Agilent Technologies). Given the reports of late non‐specific amplification in N gene assays of the US CDC 2019‐nCoV Kit (Jung *et al*., 2020; Waggoner *et al*., 2020; Won *et al*., 2020; Jaeger *et al*., 2021), a *C*_t_ cut‐off of 38 was determined to be optimal to avoid nonspecific amplification products from influencing the interpretation of test results. For simplicity, the average *C*_t_ values of both the N1 and N2 assays of the US CDC 2019‐nCoV Kit were used for comparison with the E and RdRP gene assays of the rTEST COVID‐19 qPCR kit.

#### rTEST multiplex and allplex

Clinical validation of the rTEST COVID‐19 qPCR Multiplex and Allplex kits was conducted using a selected set of 30 SARS‐CoV‐2 positive samples and 30 SARS‐CoV‐2 negative samples. The samples were thawed to re‐extract RNA using the RNAdvance Viral Kit (Cat. No. C63510; Beckman Coulter) and the Biomek i5 Automated Workstation (Beckman Coulter) and then the same index test (RdRP assay of vDetect v.1) was performed and compared with the original results using the same test to assess for RNA degradation. The new index test results served as the benchmark to assess performance of both the rTEST COVID‐19 qPCR Multiplex and Allplex kits.

#### rTEST rapid

Clinical performance of the rTEST COVID‐19 qPCR Rapid kit was conducted using a selected set of 105 SARS‐CoV‐2 positive samples and 94 SARS‐CoV‐2 negative samples. All analysed samples were obtained by gargling and were thawed before analysis. Samples for rTEST Rapid were processed and inactivated as described above. At the same time, the presence of viral RNA was detected by re‐extraction of RNA using the RNAdvance Viral Kit (Cat. No. C63510; Beckman Coulter) and the Biomek i5 Automated Workstation (Beckman Coulter) and then the index test (rTEST Allplex) was performed and compared with the results obtained without RNA extraction. The new index test results served as the benchmark to assess performance of the rTEST COVID‐19 qPCR Rapid kit.

#### rTEST COVID‐19/FLU

For SARS‐CoV‐2, the evaluation was performed on the same selected set of 38 positive and 54 negative clinical samples as used above for the vDetect and rTEST clinical validations. Regarding IAV and IBV, the evaluation was performed on a selected set of 52 and 37 clinical samples of patients with IAV and IBV, respectively, that were provided by the National Influenza Centre, National Public Health Authority of Slovak Republic in Bratislava (Bratislava, Slovakia). RNA was extracted from nasopharyngeal samples using the RNAdvance Viral Kit (Cat. No. C63510; Beckman Coulter) and the Biomek i5 Automated Workstation (Beckman Coulter). Samples were exposed to one freeze‐thaw cycle before RNA extraction. Both the index test and rTEST assays for IAV and IBV yielded a negative result for one sample so this was not included in further analyses and is not illustrated in the data.

With the exception of the independent validation at Rockefeller University, all samples used in clinical validations were previously confirmed by a reference method used for routine testing by regional public health authorities of the Slovak Republic. Testing of these selected set of samples was performed by the Biomedical Research Center, Institute of Virology, Slovak Academy of Sciences (BMC‐SAS). An experimenter, who was blinded to the sample classification, performed both the index test and evaluation test (e.g. vDetect, rTEST) in parallel and therefore the results from the index test did not influence the interpretation or outcome of the evaluation test. The experimenter used a prespecified criterion to interpret the test results for both the index and evaluation tests. All samples were processed and tested in a timely manner to minimize the effects of RNA degradation. Unless noted otherwise, *C*_t_ = 40 was selected as the cut‐off to determine positive and negative samples.

## Author contributions

PC, BK, MR, RH and EDP conceptualized and planned the study. VK conducted bioinformatic analyses for SARS‐CoV‐2 and influenza A and B primer/probe design and inclusivity and developed pipelines for filtering influenza sequences. PC and VK designed primers/probes. PP, SB, IC and MC synthesized and purified all oligonucleotides. DR, TS and TS optimized reaction conditions for vDetect v1 (HighQu master mix). KBur., AS and NV optimized reaction conditions using gel electrophoresis for vDetec v2 (Agilient Brilliant III master mix) and rTEST (Solis Biodyne SOLIScript^®^ 1‐step CoV Kit). MR, RH and DD optimized primer/probe sets on positive controls, performed analytical LoD experiments, and optimized reaction conditions using RT‐qPCR. BK, KBor., VČ, MS, ML, VV, SFH, LL, IK, JK performed wet‐lab cross reactivity tests, clinical validations and analysed clinical data. KM and TT conducted the independent validation at Rockefeller University and analysed the data. EDP, MM, RH, VK, BK and PC verified the underlying data. EDP, PC, BK, MR, KBor., RH, KBur., AS and NV analysed and interpreted the data, prepared figures and wrote the manuscript. All authors had full access to the data, provided critical comments and feedback on the manuscript and accept responsibility to submit the manuscript for publication.

## Conflict of interests

MR, RH, EDP, VK, PP, SB, IC, MC, KBur., AS, NV, DD and PC are employees of MultiplexDX, a biotechnology company which has commercialized the vDetect and rTEST kits described in this manuscript (https://www.multiplexdx.com/#products, MultiplexDX, s.r.o., Bratislava, Slovakia). PC, EDP, MR and RH are inventors and MultiplexDX, s.r.o is the assignee on EPO and PCT patent application filed that are related to the technology and kits outlined in the manuscript. BK is a Head of the Department of Virus Ecology, Institute of Virology, Biomedical Research Center of the Slovak Academy of Sciences (BMC SAS). BMC SAS has entered into a collaboration with MultiplexDX, s.r.o. for development and validation of RT‐qPCR tests for routine detection of SARS‐CoV‐2 and influenza that are described in this study. All other authors declare no competing interests.

## Supporting information


**Fig. S1.** Optimization of vDetect COVID‐19 qPCR kits. (A) Heatmap shows the optimization of reverse transcription (RT) and annealing temperatures for the HighQu 1Step RT qPCR Probe ROX L Kit using RT‐qPCR. (B) Heatmap displays the parameters optimized for the Agilent Brilliant III Ultra‐Fast QRT‐PCR Master Mix using PCR followed by gel electrophoresis. (C) Comparison of three different thermal profiles identified as being beneficial by PCR/gel electrophoresis (see Fig. S1B) using RT‐qPCR. (D) Assessment of higher RT concentration. (E) Evaluation of analytical sensitivity (limit of detection) for E and RdRP assays of the vDetect v.2 COVID‐19 RT‐qPCR test. A/E, annealing/extension; *C*_t_, cycle threshold; E, envelope gene; D, denaturation; DTT, dithiothreitol; ID, initial denaturation; ND, not detected within 45 cycles; NTC, no template control; RdRP, RNA‐dependent RNA polymerase.
**Fig. S2.** Optimization of the room‐temperature stable rTEST COVID‐19 qPCR kit. (A) Heatmap shows the optimization of thermocycling parameters for the SOLIS BioDyne SOLIScript® 1‐step CoV Kit using PCR followed by gel electrophoresis. (B) Comparison of four different thermal profiles using RT‐qPCR. (C) Plot shows the performance of various RdRP gene probes with (open bars) and without (closed bars) internal quenchers on amplification (left axis, whisker plots illustrating *C*_t_ values) and normalized fluorescence (right axis, bar graphs showing Δ*R* values). The standard probe (P2) is shown in dark gray, the best probe (P8) is shown in turquoise, other probes are shown in light gray. (D) Graph depicts comparison of E gene probes on amplification threshold. The standard probe (P1) is shown as black symbols, the best probe (P1P2) is shown as magenta symbols, other probes are shown as light gray symbols. A/E, annealing/extension; *C*_t_, cycle threshold; E, envelope gene; D, denaturation; RdRP, RNA‐dependent RNA polymerase; Δ*R*, normalized fluorescent intensity.
**Fig. S3.** Analytical sensitivity and clinical validation of rTEST COVID‐19 qPCR kit. A) Analytical sensitivity of the singleplex E, RdRP, and RNase P assays in the rTEST COVID‐19 qPCR kit. C, D) Clinical performance of the RdRP gene (C) and E gene (D) assays in the rTEST COVID‐19 qPCR kit compared to an index test (vDetect v.1) used in routine clinical practice. The dotted lines (*C*_t_ = 45) and shaded areas indicate samples that were not detected by either the evaluation test, index test, or both tests. E) Independent validation of rTEST COVID‐19 qPCR kit compared with US CDC conducted at Rockefeller University. The solid lines at *C*_t_ ≥ 38 indicate the cut‐off threshold for both index and evaluation tests to classify samples as positive or negative. The dotted lines (*C*_t_ = 45) and shaded areas indicate samples that were not detected by either the evaluation test, index test, or both tests. *C*_t_, cycle threshold; E, envelope gene; N, nucleocapsid gene; ND, not detected within 45 cycles; NTC, no template control; RdRP, RNA‐dependent RNA polymerase.
**Fig. S4.** Analytical sensitivity and clinical validation of rTEST COVID‐19 qPCR Multiplex kit. (A) Graph depicts the analytical sensitivity of the multiplexed E and RNase P assay (circle symbols) and multiplexed RdRP and RNase P assay (square symbols) in the rTEST COVID‐19 qPCR Multiplex kit. (B) Clinical performance of the rTEST COVID‐19 qPCR Multiplex kit. The dotted lines (*C*_t_ = 45) and shaded areas indicate samples that were not detected by either the evaluation test, index test, or both tests. *C*_t_, cycle threshold; E, envelope gene; ND, not detected within 45 cycles; NTC, no template control; RdRP, RNA‐dependent RNA polymerase.
**Fig. S5.** Bioinformatic analysis of mutation frequency in influenza A sequences. (A) Box and whisker plots display total number of point mutations in influenza A (IAV) H1N1 sequences for PB2 segment (top row), PB1 segment (middle row), PA segment 3 (bottom row) separated into year: 2018 (left column), 2019 (middle column), and 2020 (right columns). Boxes display the interquartile range, whiskers the min and max counts, and symbols the outliers. (B) Box and whisker plots display total number of point mutations in influenza A (IAV) H3N2 sequences for PB2 segment (top row), PB1 segment (middle row), PA segment 3 (bottom row) separated into year: 2018 (left column), 2019 (middle column), and 2020 (right columns). Boxes display the interquartile range, whiskers the min and max counts, and symbols the outliers.
**Fig. S6.** Bioinformatic analysis of mutation frequency in influenza B sequences. (A) Box and whisker plots display total number of point mutations in influenza B (IBV) Victoria sequences for PB2 segment (top row), PB1 segment (middle row), PA segment 3 (bottom row) separated into year: 2018 (left column), 2019 (middle column), and 2020 (right columns). Boxes display the interquartile range, whiskers the min and max counts, and symbols the outliers. B) Box and whisker plots display total number of point mutations in influenza B (IBV) Yamagata sequences for PB2 segment (top row), PB1 segment (middle row), PA segment 3 (bottom row) separated into year: 2018 (left column), 2019 (middle column), and 2020 (right columns). Boxes display the interquartile range, whiskers the min and max counts, and symbols the outliers.
**Fig. S7.** Bioinformatic analysis of unknown bases in influenza A sequences. (A) Box and whisker plots display total number of unknown bases in influenza A (IAV) H1N1 sequences for PB2 segment (top row), PB1 segment (middle row), PA segment 3 (bottom row) separated into year: 2018 (left column), 2019 (middle column), and 2020 (right columns). Boxes display the interquartile range, whiskers the min and max counts, and symbols the outliers. (B) Box and whisker plots display total number of unknown bases in influenza A (IAV) H3N2 sequences for PB2 segment (top row), PB1 segment (middle row), PA segment 3 (bottom row) separated into year: 2018 (left column), 2019 (middle column), and 2020 (right columns). Boxes display the interquartile range, whiskers the min and max counts, and symbols the outliers.
**Fig. S8.** Bioinformatic analysis of unknown bases in influenza B sequences. (A) Box and whisker plots display total number of unknown bases in influenza B (IBV) Victoria sequences for PB2 segment (top row), PB1 segment (middle row), PA segment 3 (bottom row) separated into year: 2018 (left column), 2019 (middle column), and 2020 (right columns). Boxes display the interquartile range, whiskers the min and max counts, and symbols the outliers. (B) Box and whisker plots display total number of unknown bases in influenza B (IBV) Yamagata sequences for PB2 segment (top row), PB1 segment (middle row), PA segment 3 (bottom row) separated into year: 2018 (left column), 2019 (middle column), and 2020 (right columns). Boxes display the interquartile range, whiskers the min and max counts, and symbols the outliers.
**Fig. S9.** Bioinformatic analysis of geographical biases for IAV and IBV. (A) Bar graphs illustrate the number of geographical areas (counts, y‐axis) that contain a given number of sequences (log10‐scaled, x‐axis) for IAV H1N1 (top row) and IAV H3N2 (bottom row) separated into year: 2018 (left column), 2019 (middle column), and 2020 (right columns). (B) Bar graphs illustrate the number of geographical areas (counts, y‐axis) that contain a given number of sequences (log_10_‐scaled, *x*‐axis) for IBV Victoria (top row) and IBV Yamagata (bottom row) separated into year: 2018 (left column), 2019 (middle column), and 2020 (right columns). Geographical regions containing more than 100 sequences (log_10_‐scaled count ≥ 2) were further investigated for clonal biases.
**Fig. S10.** Conservation of IAV sequences. (A) Sliding window plots show the averaged max probability of base assignment at each position for IAV H1N1 PB2 segment (top panel), PB1 segment (middle panel), and PA segment (bottom panel). (B) Sliding window plots show the averaged max probability of base assignment at each position for IAV H3N2 PB2 segment (top panel), PB1 segment (middle panel), and PA segment (bottom panel). Sliding windows have a 30 bp width with a 10 bp shift.
**Fig. S11.** Conservation of IBV sequences. (A) Sliding window plots show the averaged max probability of base assignment at each position for IBV Victoria PB2 segment (top panel), PB1 segment (middle panel), and PA segment (bottom panel). (B) Sliding window plots show the averaged max probability of base assignment at each position for IBV Yamagata PB2 segment (top panel), PB1 segment (middle panel), and PA segment (bottom panel). Sliding windows have a 30 bp width with a 10 bp shift.
**Fig. S12.** Optimization and analytical sensitivity of rTEST COVID‐19/FLU qPCR kit. (A) Plot shows the performance of single and dual probes for IAV on amplification (left axis, whisker plots illustrating *C*_t_ values) and normalized fluorescence (right axis, bar graphs showing Δ*R* values). The best probe (P1.2) is shown in yellow, while other probes are shown in gray. (B) Plot shows the performance of single and dual probes for IBV on amplification (left axis, whisker plots illustrating *C*_t_ values) and normalized fluorescence (right axis, bar graphs showing Δ*R* values). The best probe (P2.2) is shown in green, while other probes are shown in gray. (C, D) Graphs depict the analytical sensitivity of the multiplexed SARS‐CoV‐2 E, IAV PB1, and RNase P assay (C) and multiplexed SARS‐CoV‐2 RdRP, IBV PA, and RNase P assay (D) in the rTEST COVID‐19/FLU qPCR kit. (E‐H) Assessment of assay inhibition due to reagent consumption using 4‐fold dilutions of samples containing all templates starting at a high viral load (5000x LoD, 10,240 copies/reaction) on cycle threshold (E, F) and normalized fluorescent intensity (G, H) of the multiplexed E, IAV, and RNase P assay (E, G) and the multiplexed RdRP, IBV, and RNase P assay (F, H). The dotted line at *C*_t_ 40 (C and D) serves as a threshold after which amplification is considered invalid. *C*_t_, cycle threshold; E, envelope gene; IAV, influenza A; IBV, influenza B; PA, polymerase acidic protein; PB1, polymerase basic 1 protein; ND, not detected within 45 cycles; NTC, no template control; RdRP, RNA‐dependent RNA polymerase; Δ*R*, normalized fluorescent intensity.
**Table S1.** Sequences of all primers and probes that were used during optimization of SARS‐CoV‐2, IAV and IBV detection.
**Table S2.** Optimization of one‐step RT‐qPCR thermal profiles and reaction mixtures.
**Table S3.** Cross‐reactivity (specificity) testing.
**Table S4.** Clinical performance of vDetect v.1 COVID‐19 RT‐qPCR kit.
**Table S5.** Clinical performance of rTEST COVID‐19 qPCR kit.
**Table S6.** Clinical performance of rTEST COVID‐19 qPCR Multiplex and Allplex kits.
**Table S7.** Clinical performance of rTEST COVID‐19 qPCR Rapid kit.
**Table S8.** Clinical performance of rTEST COVID‐19/FLU qPCR kit.
