# Supplementary material for: Sequential development of several RT‐qPCR tests using LNA nucleotides and dual probe technology to differentiate SARS‐CoV‐2 from influenza A and B
Source: Microb Biotechnol. 2022 Mar 22;15(7):1995–2021. doi: 10.1111/1751-7915.14031 (PMC9111289; doi:10.1111/1751-7915.14031)
Supplement: Supplementary file 1 — Fig. S1. Optimization of vDetect COVID‐19 qPCR kits. (A) Heatmap shows the optimization of reverse transcription (RT) and annealing temperatures for the HighQu 1Step RT qPCR Probe ROX L Kit using RT‐qPCR. (B) Heatmap displays the parameters optimized for the Agilent Brilliant III Ultra‐Fast QRT‐PCR Master Mix using PCR followed by gel electrophoresis. (C) Comparison of three different thermal profiles identified as being beneficial by PCR/gel electrophoresis (see Fig. S1B) using RT‐qPCR. (D) Assessment of higher RT concentration. (E) Evaluation of analytical sensitivity (limit of detection) for E and RdRP assays of the vDetect v.2 COVID‐19 RT‐qPCR test. A/E, annealing/extension; C t, cycle threshold; E, envelope gene; D, denaturation; DTT, dithiothreitol; ID, initial denaturation; ND, not detected within 45 cycles; NTC, no template control; RdRP, RNA‐dependent RNA polymerase. Fig. S2. Optimization of the room‐temperature stable rTEST COVID‐19 qPCR kit. (A) Heatmap shows the optimization of thermocycling parameters for the SOLIS BioDyne SOLIScript® 1‐step CoV Kit using PCR followed by gel electrophoresis. (B) Comparison of four different thermal profiles using RT‐qPCR. (C) Plot shows the performance of various RdRP gene probes with (open bars) and without (closed bars) internal quenchers on amplification (left axis, whisker plots illustrating C t values) and normalized fluorescence (right axis, bar graphs showing ΔR values). The standard probe (P2) is shown in dark gray, the best probe (P8) is shown in turquoise, other probes are shown in light gray. (D) Graph depicts comparison of E gene probes on amplification threshold. The standard probe (P1) is shown as black symbols, the best probe (P1P2) is shown as magenta symbols, other probes are shown as light gray symbols. A/E, annealing/extension; C t, cycle threshold; E, envelope gene; D, denaturation; RdRP, RNA‐dependent RNA polymerase; ΔR, normalized fluorescent intensity. Fig. S3. Analytical sensitivity and clinical [file MBT2-15-1995-s001.docx]

**Supporting information**

Sequential development of several RT-qPCR tests using LNA nucleotides and dual probe technology to differentiate SARS-CoV-2 from influenza A and B

Monika Radvánszka, Evan D. Paul, Roman Hajdu, Kristína Boršová, Viera Kováčová, Piotr Putaj, Stanislava Bírová, Ivana Čirková, Martin Čarnecký, Katarína Buranovská, Adrián Szobi, Nina Vojtaššáková, Diana Drobná, Viktória Čabanová, Monika Sláviková, Martina Ličková, Veronika Vaňová, Sabína Fumačová Havlíková, Ľubomíra Lukáčiková, Ivana Kajanová, Juraj Koči, Diana Rusňáková, Tatiana Sedláčková, Klaas E.A. Max, Thomas Tuschl, Tomáš Szemes, Boris Klempa, Pavol Čekan

**Supplementary Figure S1.**

**
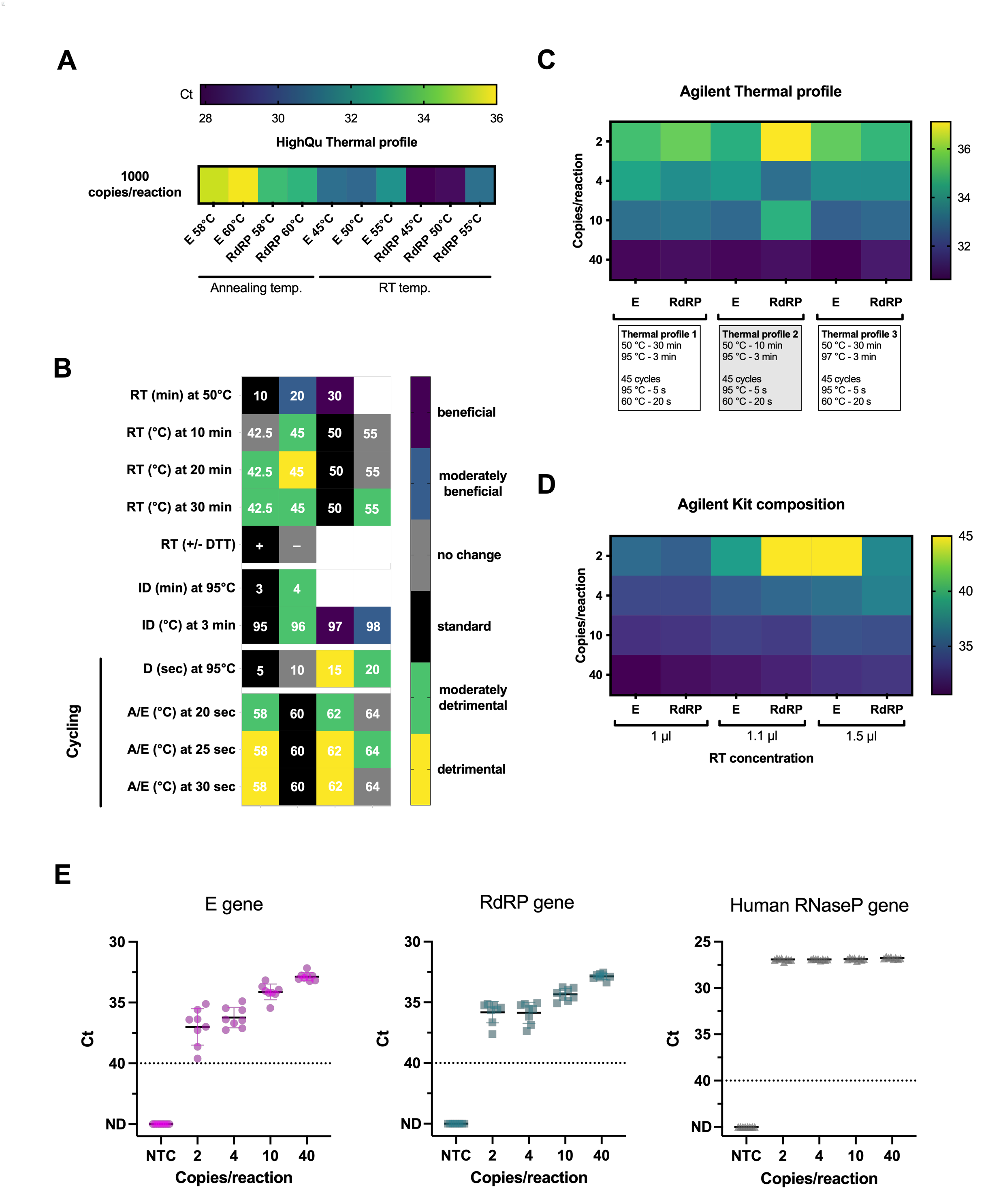
**

**Supplementary Figure S1. Optimization of vDetect COVID-19 qPCR kits.**

**A**) Heatmap shows the optimization of reverse transcription (RT) and annealing temperatures for the HighQu 1Step RT qPCR Probe ROX L Kit using RT-qPCR. **B**) Heatmap displays the parameters optimized for the Agilent Brilliant III Ultra-Fast QRT-PCR Master Mix using PCR followed by gel electrophoresis. **C**) Comparison of three different thermal profiles identified as being beneficial by PCR/gel electrophoresis (see **Figure S1B**) using RT-qPCR. **D**) Assessment of higher RT concentration. **E**) Evaluation of analytical sensitivity (limit of detection) for E and RdRP assays of the vDetect v.2 COVID-19 RT-qPCR test. A/E, annealing/extension; Ct, cycle threshold; E, envelope gene; D, denaturation; DTT, dithiothreitol; ID, initial denaturation; ND, not detected within 45 cycles; NTC, no template control; RdRP, RNA-dependent RNA polymerase.

**Supplementary Figure S2.**

**
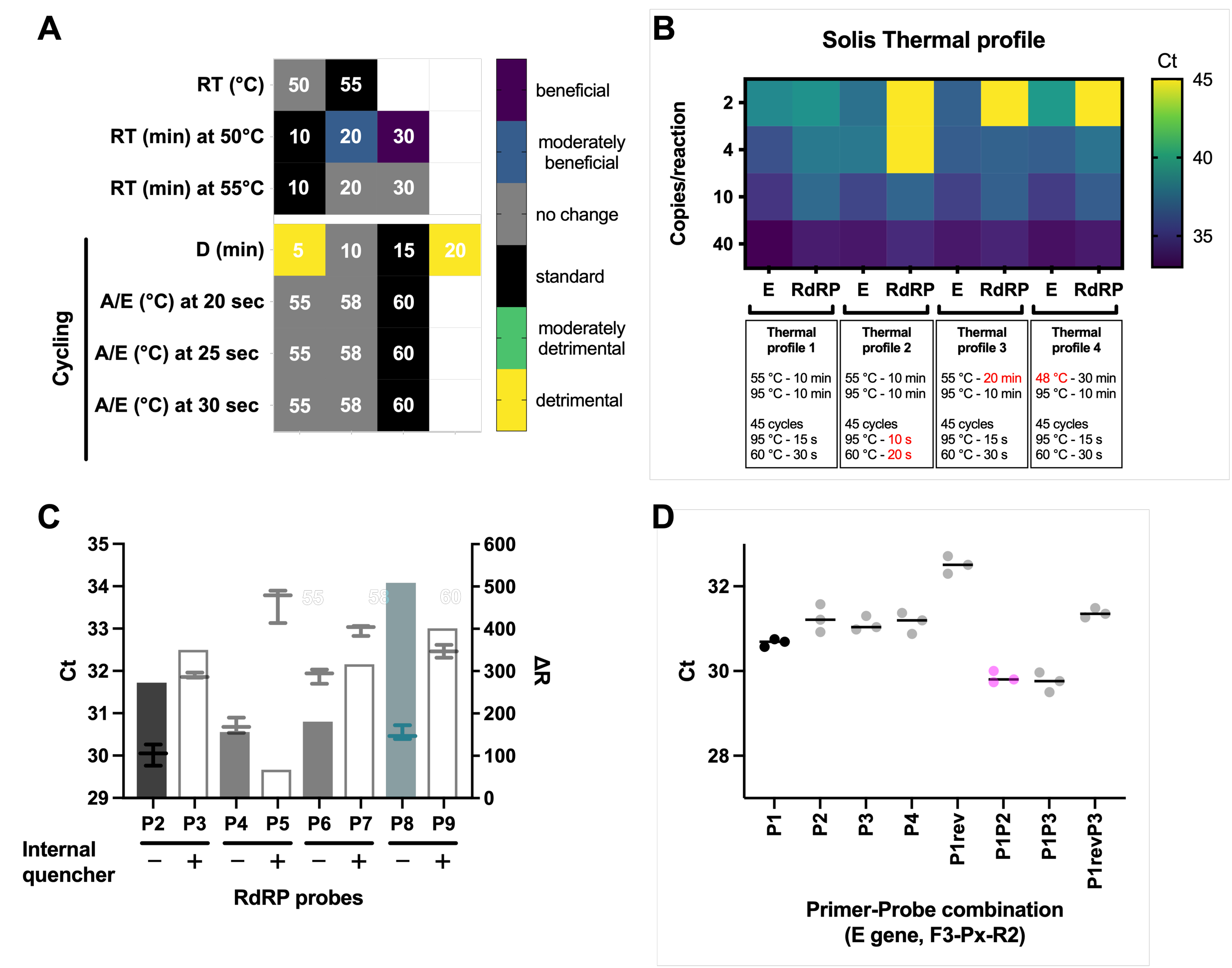
**

**Supplementary Figure S2. Optimization of the room-temperature stable rTEST COVID-19 qPCR kit.**

**A**) Heatmap shows the optimization of thermocycling parameters for the SOLIS BioDyne SOLIScript® 1-step CoV Kit using PCR followed by gel electrophoresis. **B**) Comparison of four different thermal profiles using RT-qPCR. **C**) Plot shows the performance of various RdRP gene probes with (open bars) and without (closed bars) internal quenchers on amplification (left axis, whisker plots illustrating Ct values) and normalized fluorescence (right axis, bar graphs showing ΔR values). The standard probe (P2) is shown in dark gray, the best probe (P8) is shown in turquoise, other probes are shown in light gray. **D**) Graph depicts comparison of E gene probes on amplification threshold. The standard probe (P1) is shown as black symbols, the best probe (P1P2) is shown as magenta symbols, other probes are shown as light gray symbols. A/E, annealing/extension; Ct, cycle threshold; E, envelope gene; D, denaturation; RdRP, RNA-dependent RNA polymerase; ΔR, normalized fluorescent intensity.

**Supplementary Figure S3.**

**
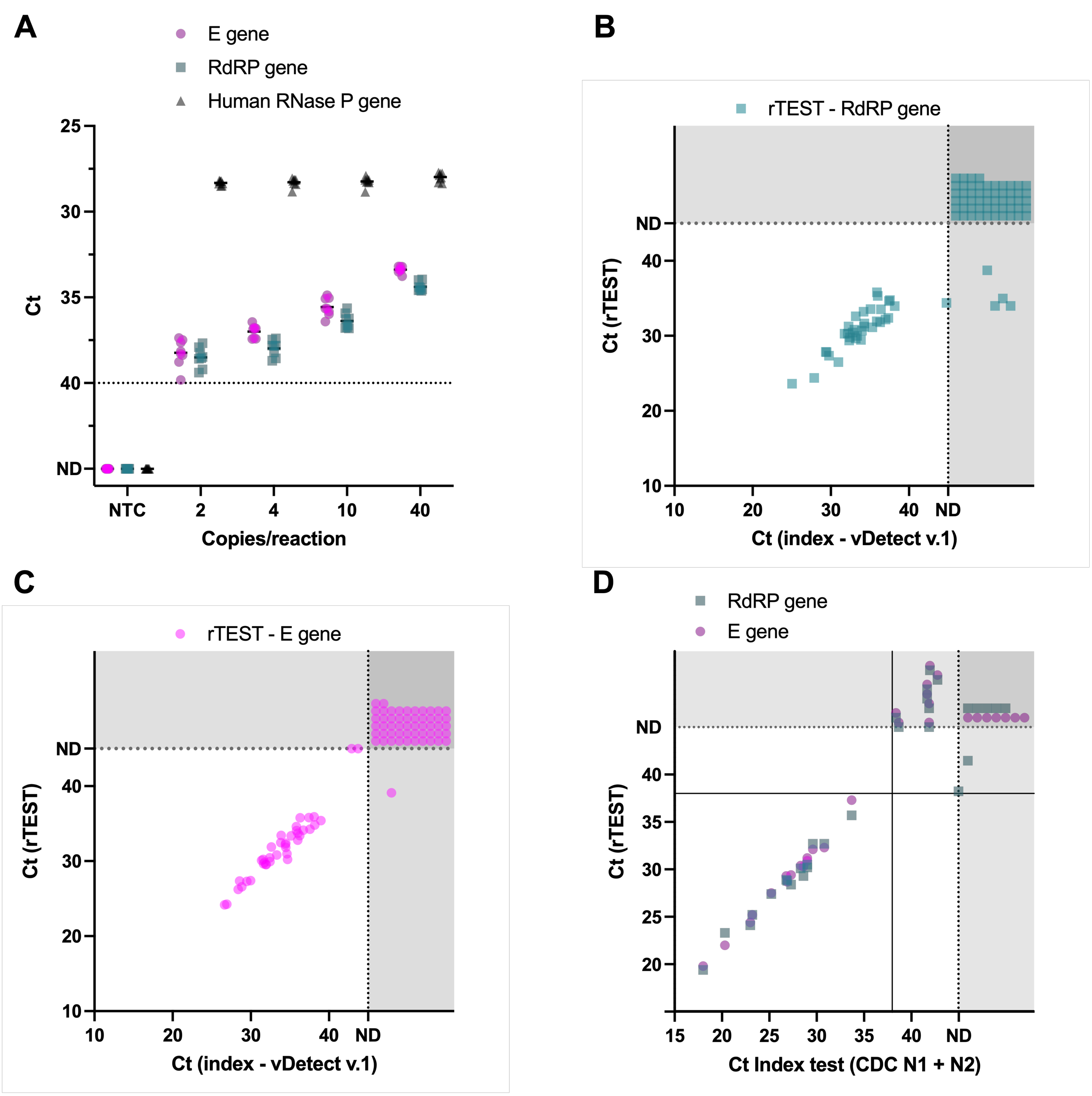
**

**Supplementary Figure S3. Analytical sensitivity and clinical validation of rTEST COVID-19 qPCR kit.**

**A**) Analytical sensitivity of the singleplex E, RdRP, and RNase P assays in the rTEST COVID-19 qPCR kit. **C**, **D**) Clinical performance of the RdRP gene (**C**) and E gene (**D**) assays in the rTEST COVID-19 qPCR kit compared to an index test (vDetect v.1) used in routine clinical practice. The dotted lines (Ct = 45) and shaded areas indicate samples that were not detected by either the evaluation test, index test, or both tests. **E**) Independent validation of rTEST COVID-19 qPCR kit compared with US CDC conducted at Rockefeller University. The solid lines at Ct ≥ 38 indicate the cut-off threshold for both index and evaluation tests to classify samples as positive or negative. The dotted lines (Ct = 45) and shaded areas indicate samples that were not detected by either the evaluation test, index test, or both tests. Ct, cycle threshold; E, envelope gene; N, nucleocapsid gene; ND, not detected within 45 cycles; NTC, no template control; RdRP, RNA-dependent RNA polymerase.

**Supplementary Figure S4.**

**
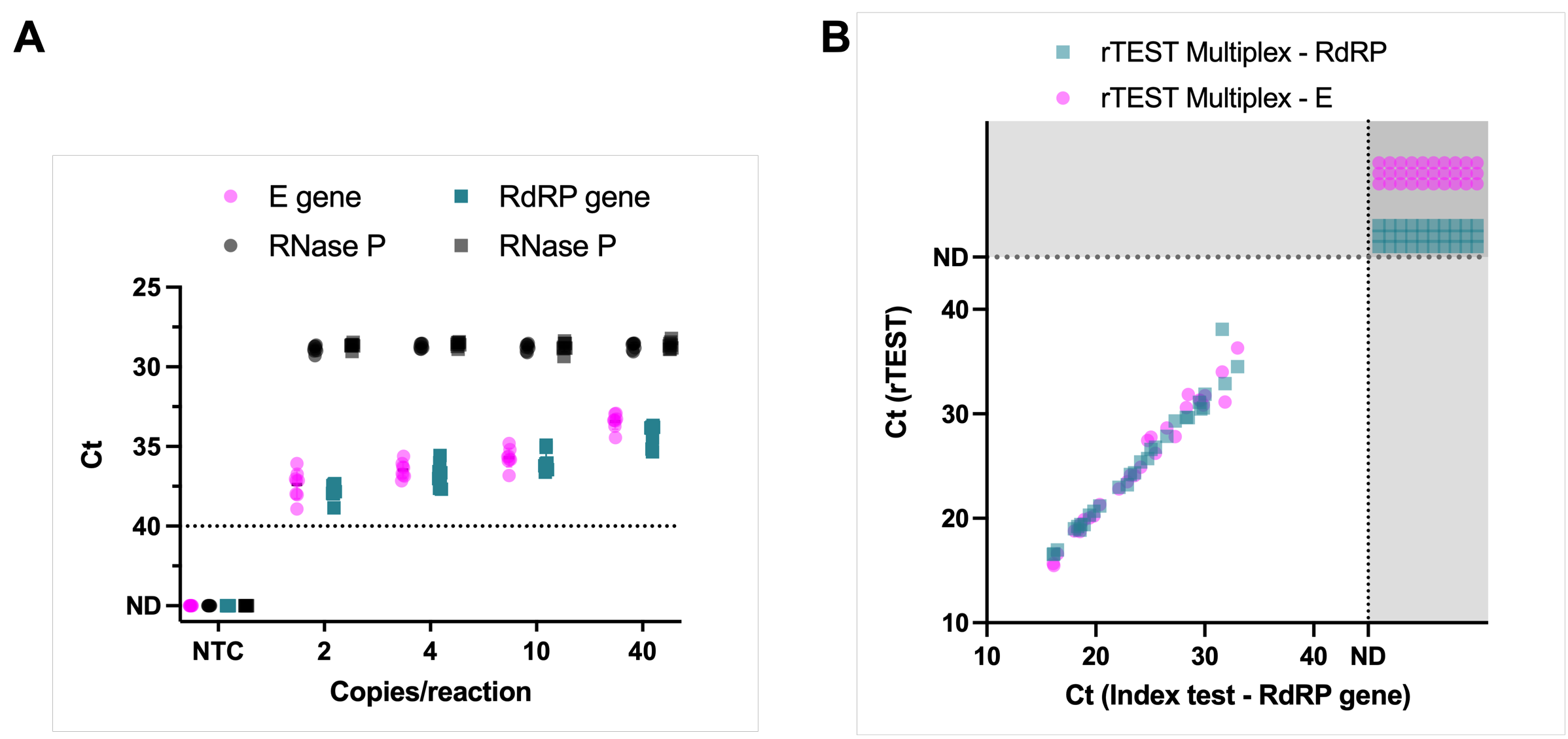
**

**Supplementary Figure S4. Analytical sensitivity and clinical validation of rTEST COVID-19 qPCR Multiplex kit.**

**A**) Graph depicts the analytical sensitivity of the multiplexed E and RNase P assay (circle symbols) and multiplexed RdRP and RNase P assay (square symbols) in the rTEST COVID-19 qPCR Multiplex kit. **B**) Clinical performance of the rTEST COVID-19 qPCR Multiplex kit. The dotted lines (Ct = 45) and shaded areas indicate samples that were not detected by either the evaluation test, index test, or both tests. Ct, cycle threshold; E, envelope gene; ND, not detected within 45 cycles; NTC, no template control; RdRP, RNA-dependent RNA polymerase.

**Supplementary Figure S5.**

**
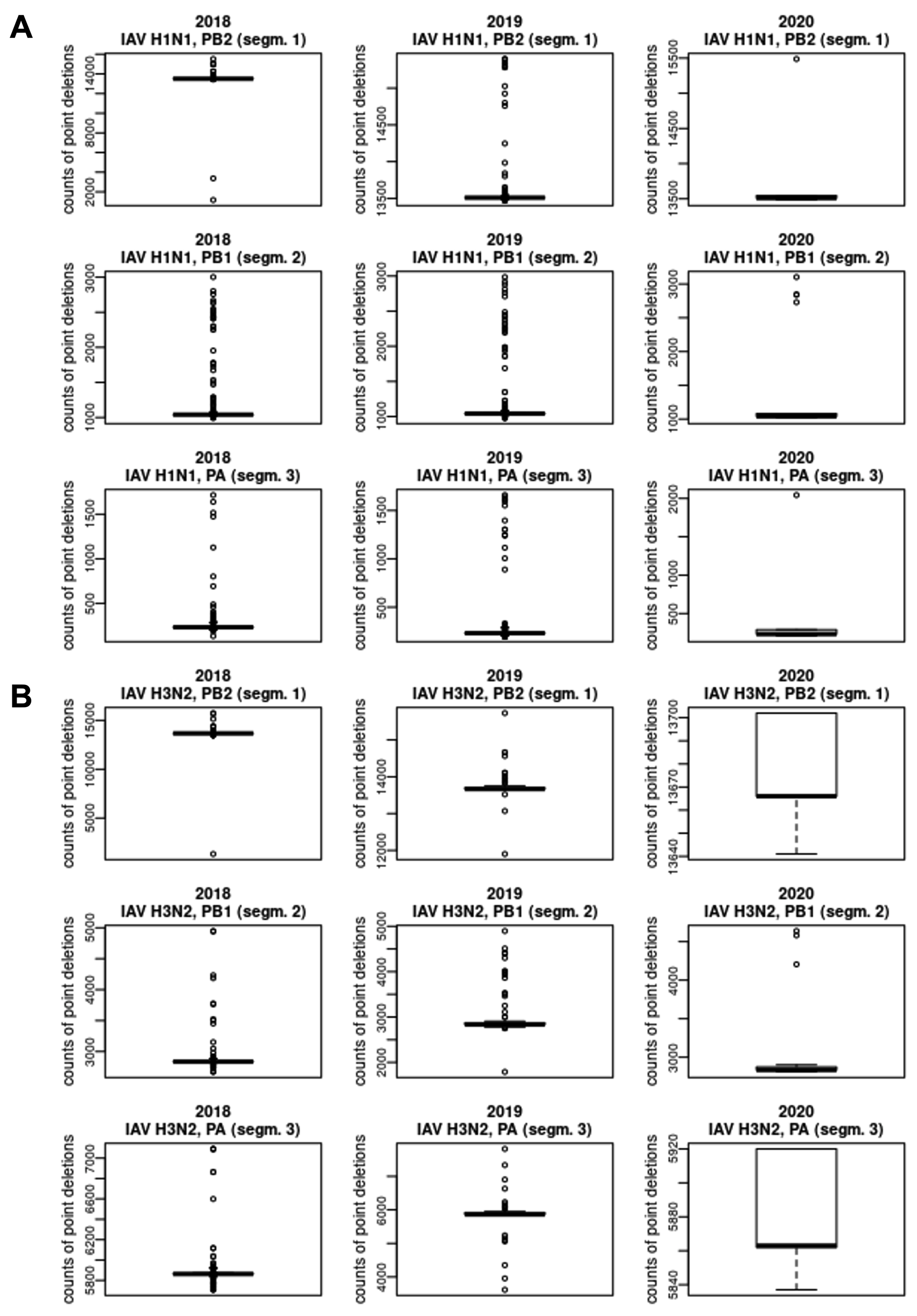
**

**Supplementary Figure S5. Bioinformatic analysis of mutation frequency in influenza A sequences.**

**A)** Box and whisker plots display total number of point mutations in influenza A (IAV) H1N1 sequences for PB2 segment (top row), PB1 segment (middle row), PA segment 3 (bottom row) separated into year: 2018 (left column), 2019 (middle column), and 2020 (right columns). Boxes display the interquartile range, whiskers the min and max counts, and symbols the outliers. **B)** Box and whisker plots display total number of point mutations in influenza A (IAV) H3N2 sequences for PB2 segment (top row), PB1 segment (middle row), PA segment 3 (bottom row) separated into year: 2018 (left column), 2019 (middle column), and 2020 (right columns). Boxes display the interquartile range, whiskers the min and max counts, and symbols the outliers.

**Supplementary Figure S6**

**
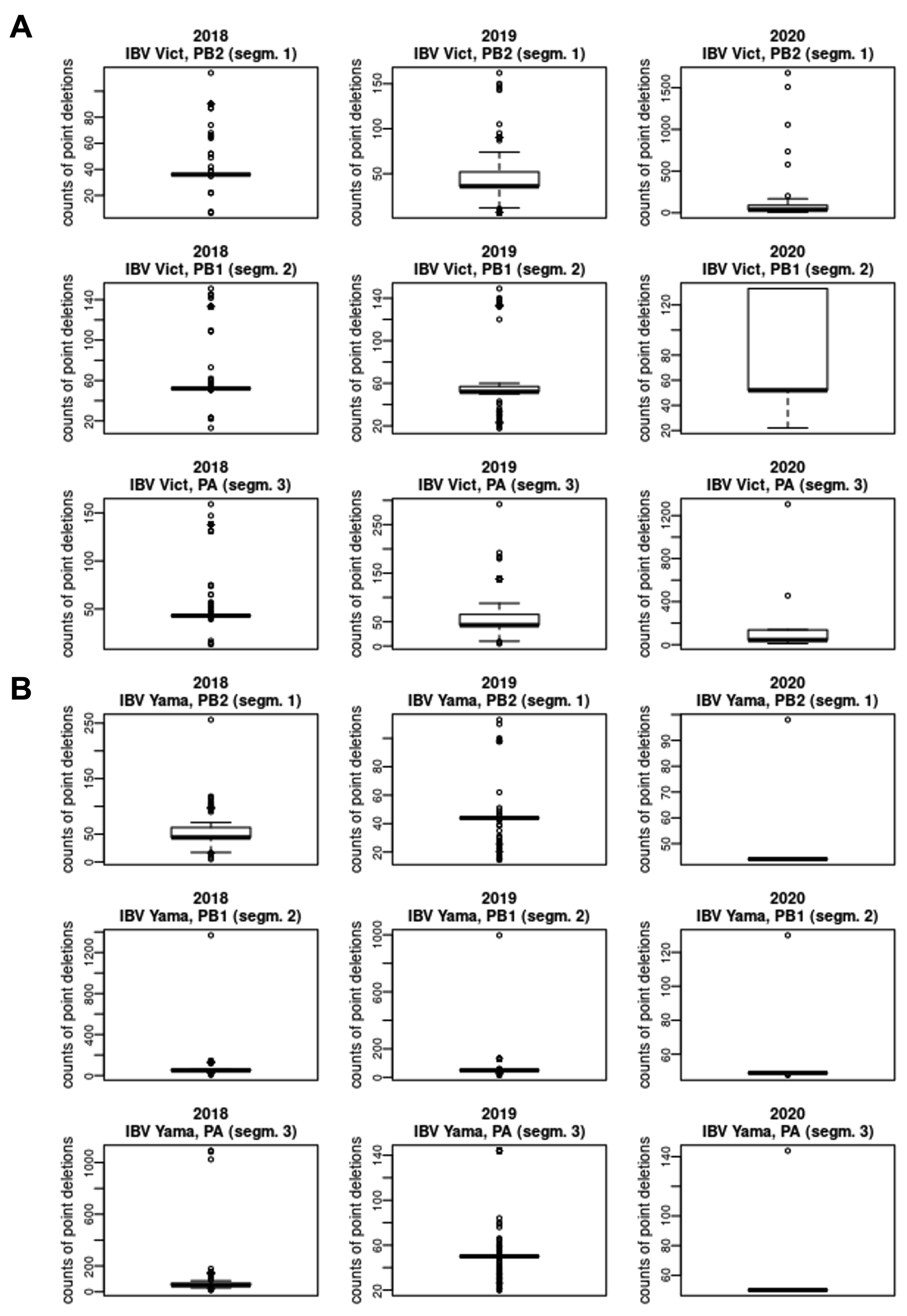
**

**Supplementary Figure S6. Bioinformatic analysis of mutation frequency in influenza B sequences.**

**A)** Box and whisker plots display total number of point mutations in influenza B (IBV) Victoria sequences for PB2 segment (top row), PB1 segment (middle row), PA segment 3 (bottom row) separated into year: 2018 (left column), 2019 (middle column), and 2020 (right columns). Boxes display the interquartile range, whiskers the min and max counts, and symbols the outliers. **B)** Box and whisker plots display total number of point mutations in influenza B (IBV) Yamagata sequences for PB2 segment (top row), PB1 segment (middle row), PA segment 3 (bottom row) separated into year: 2018 (left column), 2019 (middle column), and 2020 (right columns). Boxes display the interquartile range, whiskers the min and max counts, and symbols the outliers.

**Supplementary Figure S7.**

**
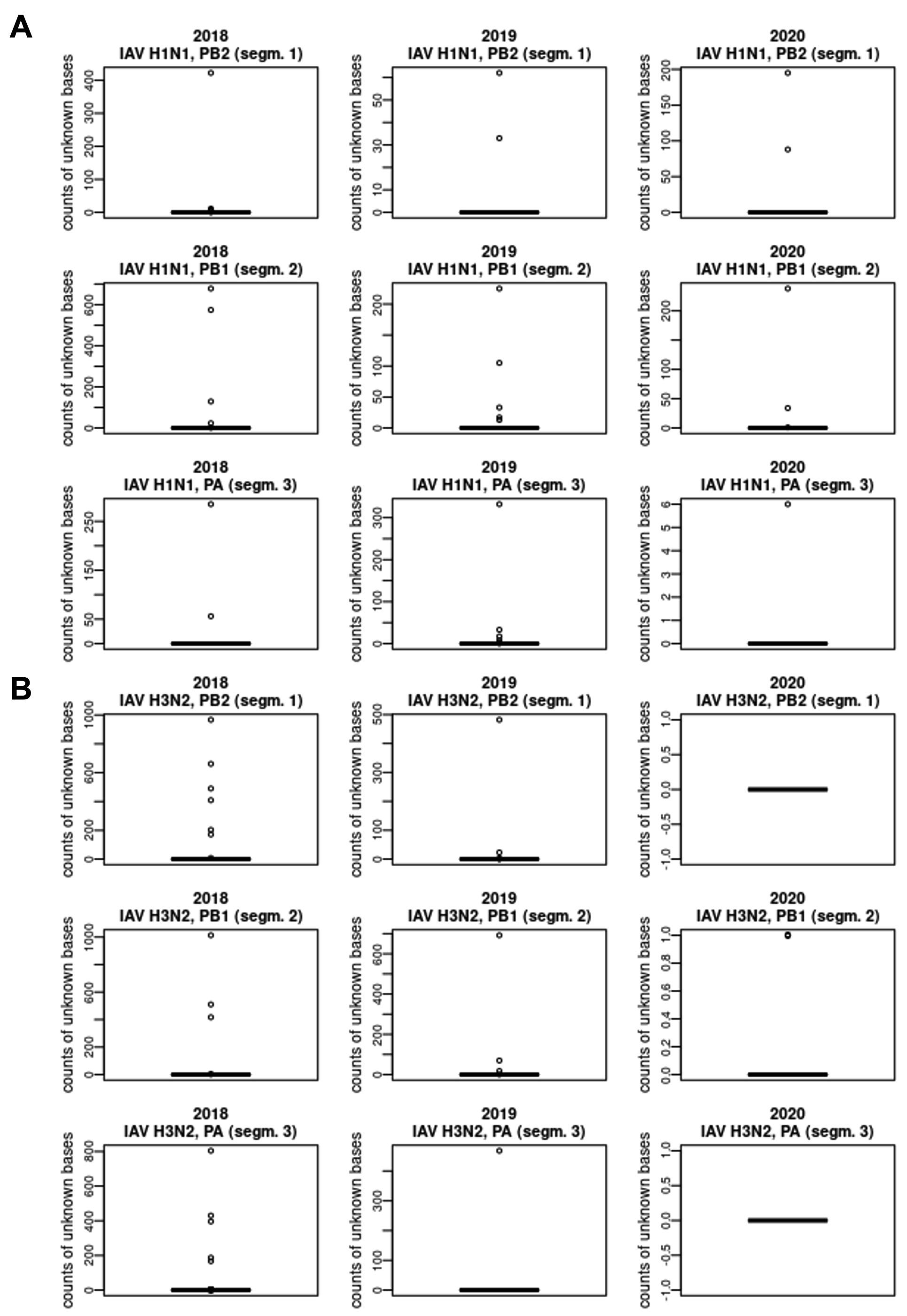
**

**Supplementary Figure S7. Bioinformatic analysis of unknown bases in influenza A sequences.**

**A)** Box and whisker plots display total number of unknown bases in influenza A (IAV) H1N1 sequences for PB2 segment (top row), PB1 segment (middle row), PA segment 3 (bottom row) separated into year: 2018 (left column), 2019 (middle column), and 2020 (right columns). Boxes display the interquartile range, whiskers the min and max counts, and symbols the outliers. **B)** Box and whisker plots display total number of unknown bases in influenza A (IAV) H3N2 sequences for PB2 segment (top row), PB1 segment (middle row), PA segment 3 (bottom row) separated into year: 2018 (left column), 2019 (middle column), and 2020 (right columns). Boxes display the interquartile range, whiskers the min and max counts, and symbols the outliers.

**Supplementary Figure S8.**

**
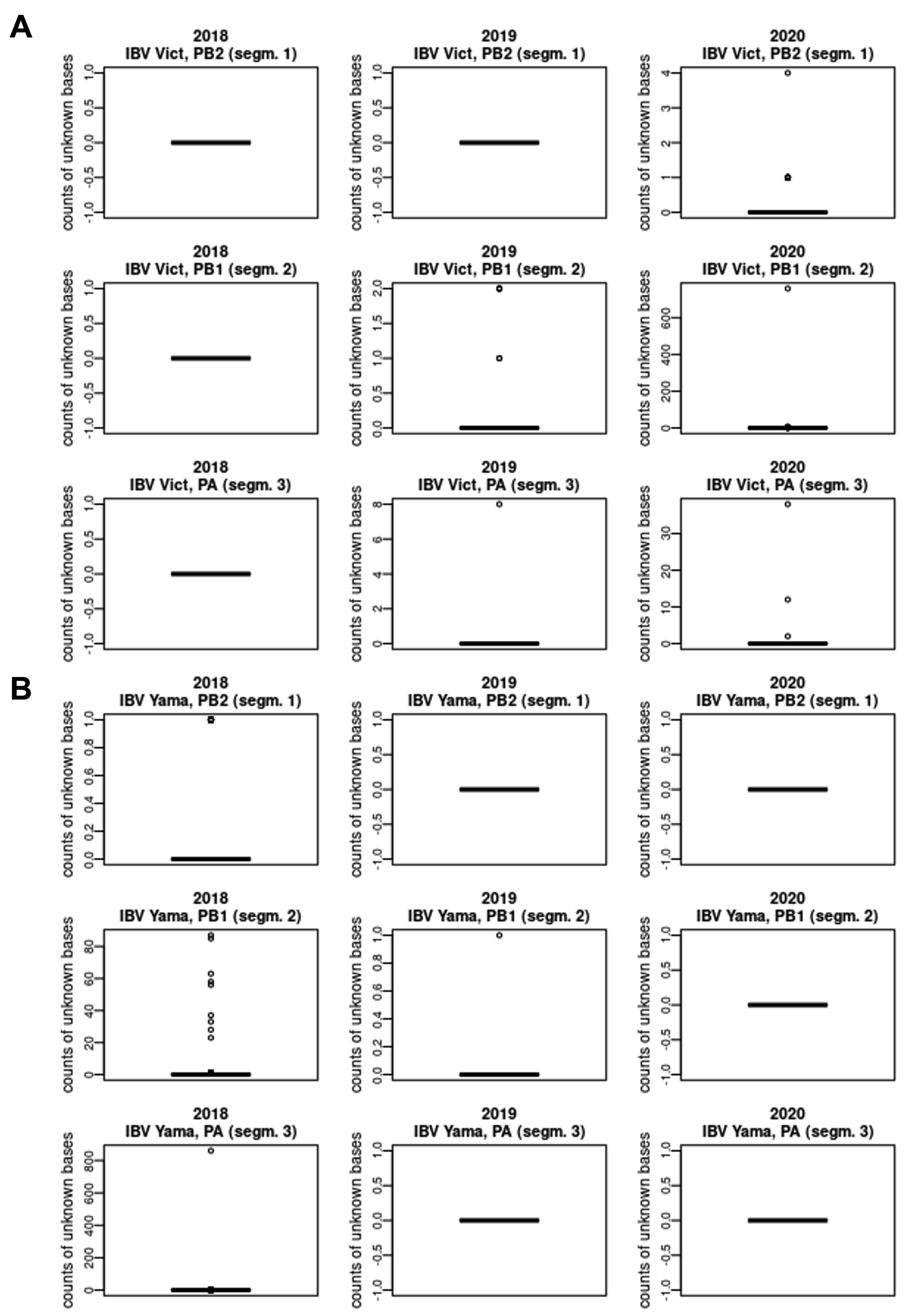
**

**Supplementary Figure S8. Bioinformatic analysis of unknown bases in influenza B sequences.**

**A)** Box and whisker plots display total number of unknown bases in influenza B (IBV) Victoria sequences for PB2 segment (top row), PB1 segment (middle row), PA segment 3 (bottom row) separated into year: 2018 (left column), 2019 (middle column), and 2020 (right columns). Boxes display the interquartile range, whiskers the min and max counts, and symbols the outliers. **B)** Box and whisker plots display total number of unknown bases in influenza B (IBV) Yamagata sequences for PB2 segment (top row), PB1 segment (middle row), PA segment 3 (bottom row) separated into year: 2018 (left column), 2019 (middle column), and 2020 (right columns). Boxes display the interquartile range, whiskers the min and max counts, and symbols the outliers.

**Supplementary Figure S9.**

**
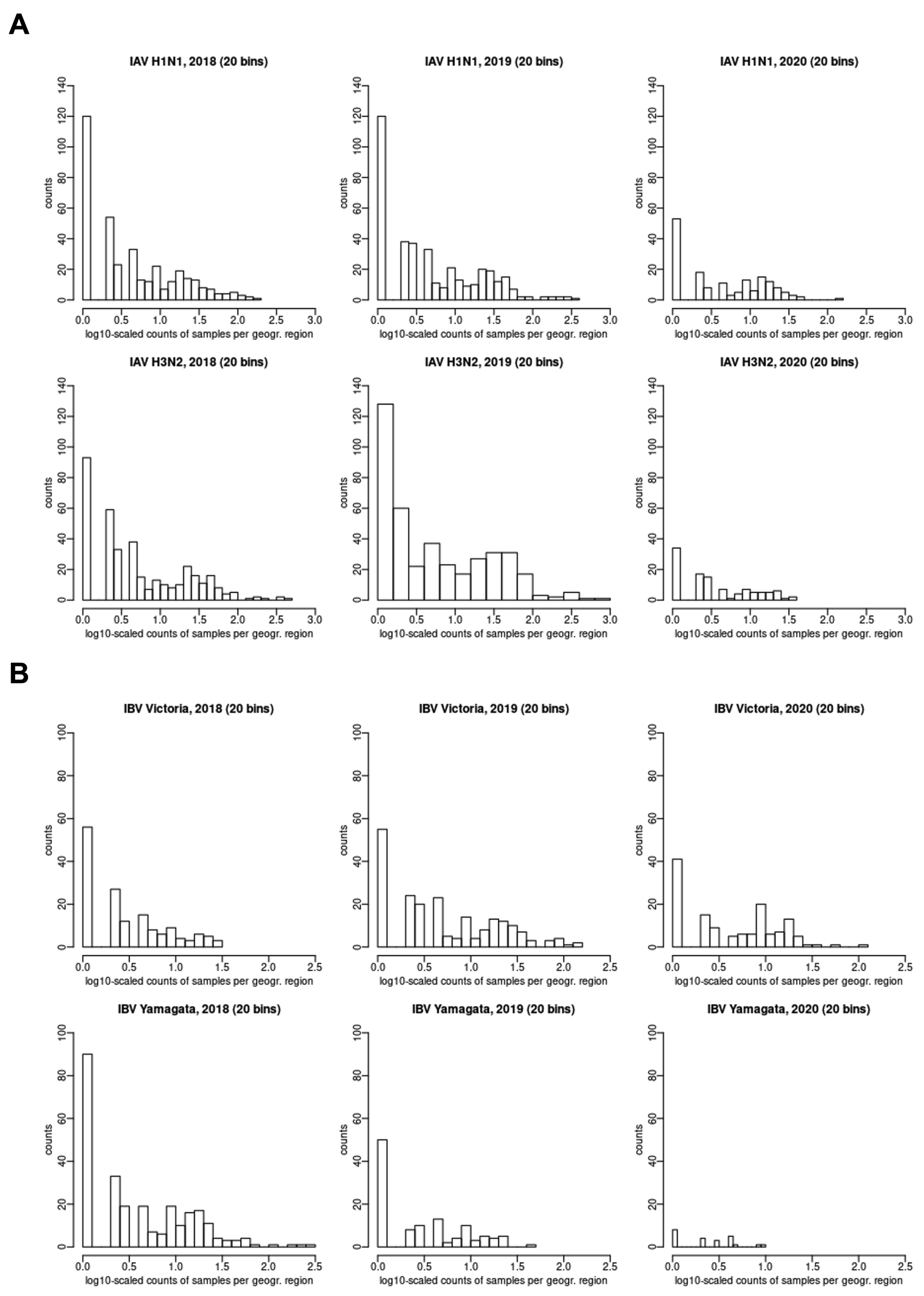
**

**Supplementary Figure S9. Bioinformatic analysis of geographical biases for IAV and IBV.**

**A)** Bar graphs illustrate the number of geographical areas (counts, y-axis) that contain a given number of sequences (log10-scaled, x-axis) for IAV H1N1 (top row) and IAV H3N2 (bottom row) separated into year: 2018 (left column), 2019 (middle column), and 2020 (right columns). **B)** Bar graphs illustrate the number of geographical areas (counts, y-axis) that contain a given number of sequences (log10-scaled, x-axis) for IBV Victoria (top row) and IBV Yamagata (bottom row) separated into year: 2018 (left column), 2019 (middle column), and 2020 (right columns). Geographical regions containing more than 100 sequences (log10-scaled count ≥ 2) were further investigated for clonal biases.

**Supplementary Figure S10.**

**
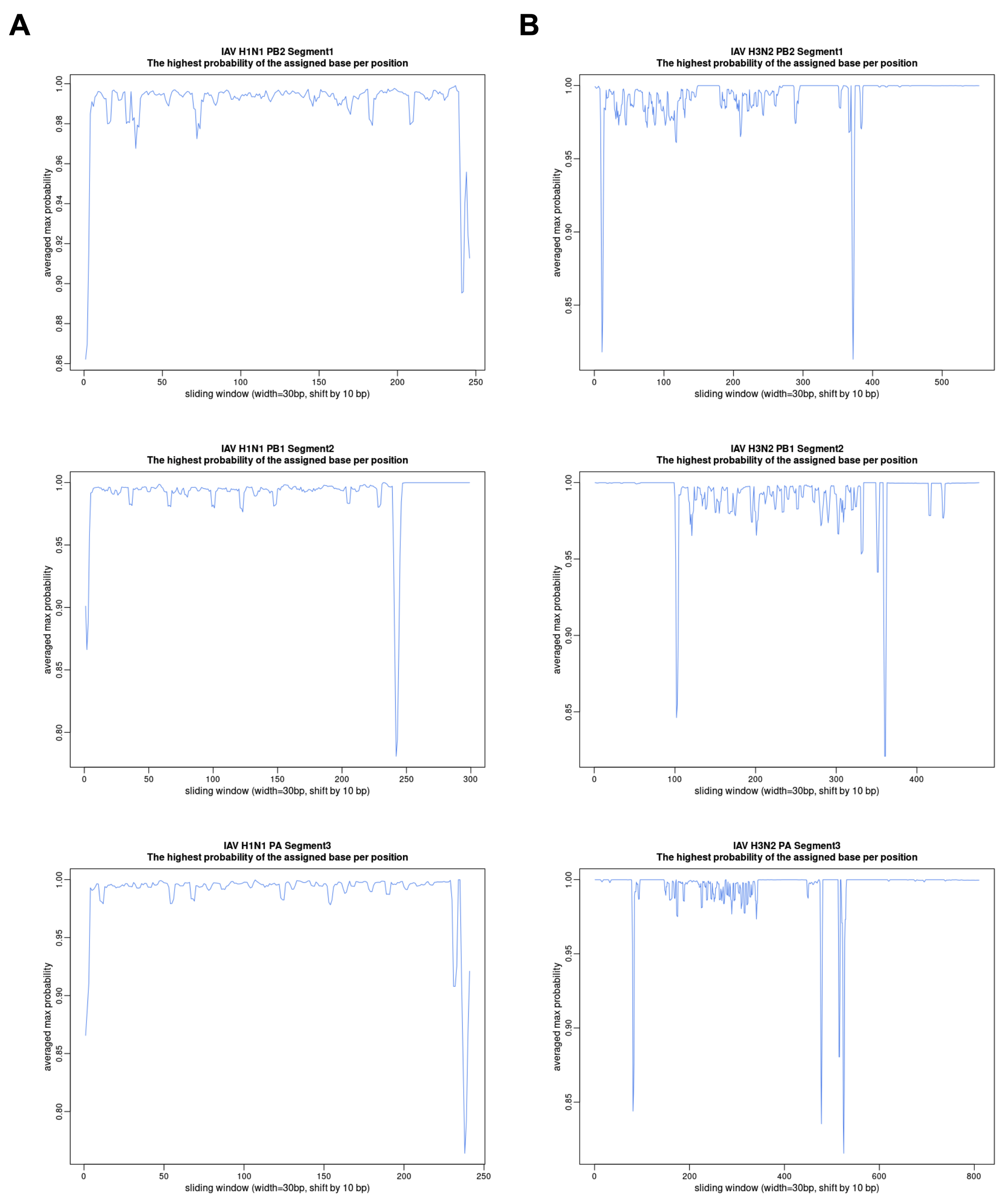
**

**Supplementary Figure S10. Conservation of IAV sequences.**

**A)** Sliding window plots show the averaged max probability of base assignment at each position for IAV H1N1 PB2 segment (top panel), PB1 segment (middle panel), and PA segment (bottom panel). **B)** Sliding window plots show the averaged max probability of base assignment at each position for IAV H3N2 PB2 segment (top panel), PB1 segment (middle panel), and PA segment (bottom panel). Sliding windows have a 30 bp width with a 10 bp shift.

**Supplementary Figure S11.**

**
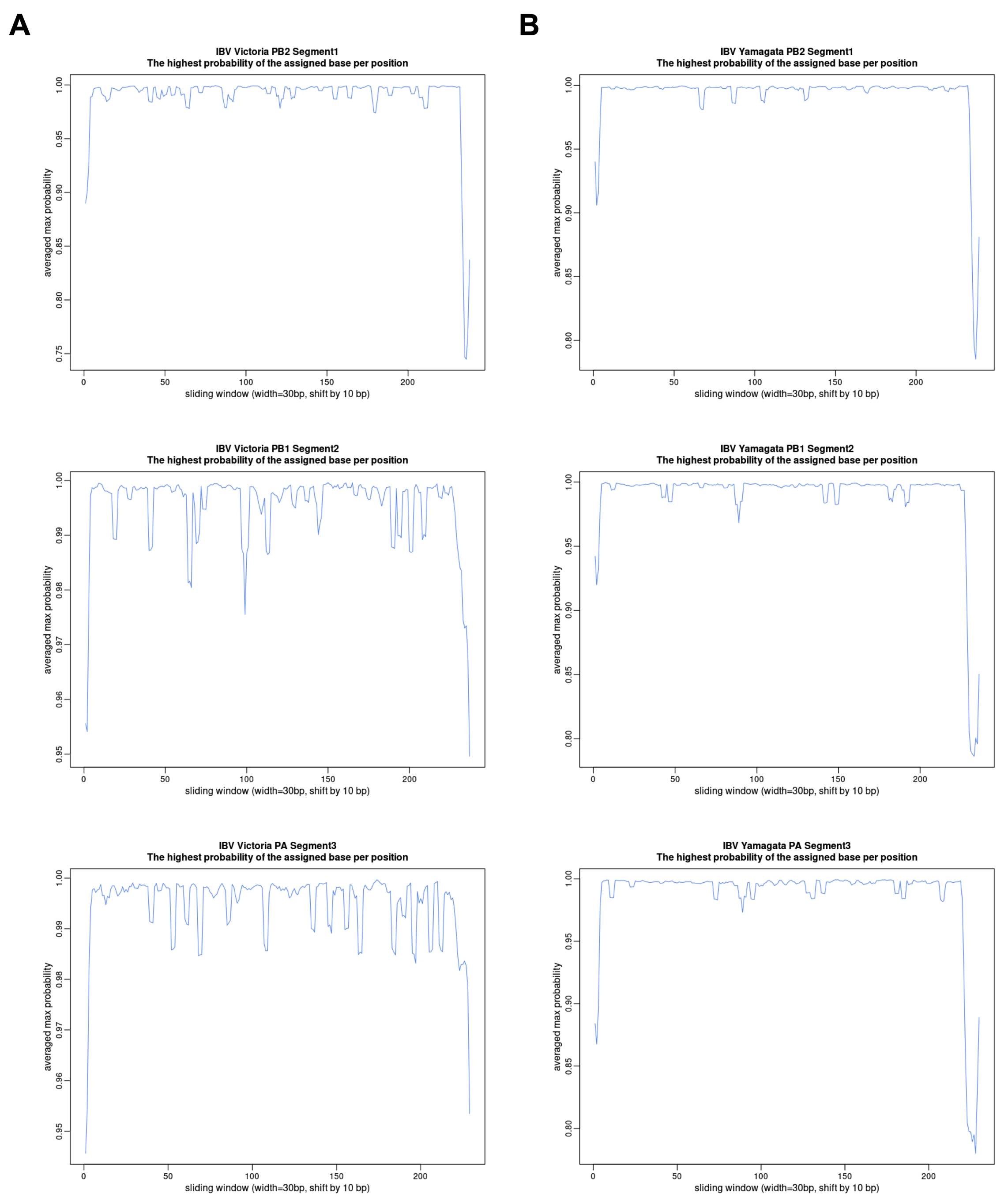
**

**Supplementary Figure S11. Conservation of IBV sequences.**

**A)** Sliding window plots show the averaged max probability of base assignment at each position for IBV Victoria PB2 segment (top panel), PB1 segment (middle panel), and PA segment (bottom panel). **B)** Sliding window plots show the averaged max probability of base assignment at each position for IBV Yamagata PB2 segment (top panel), PB1 segment (middle panel), and PA segment (bottom panel). Sliding windows have a 30 bp width with a 10 bp shift.

**Supplementary Figure S12.**


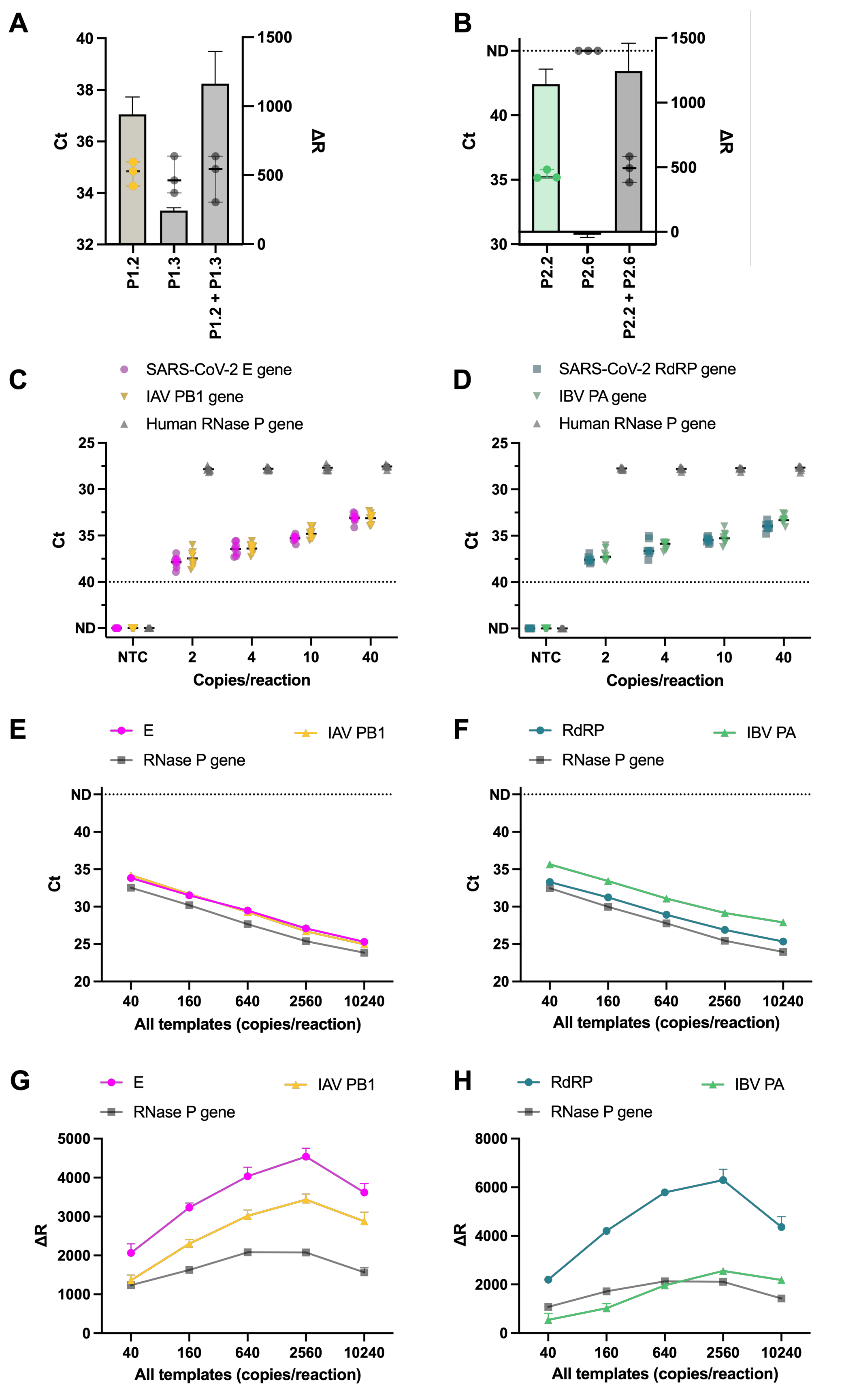


**Supplementary Figure S12. Optimization and analytical sensitivity of rTEST COVID-19/FLU qPCR kit.**

(**A**) Plot shows the performance of single and dual probes for IAV on amplification (left axis, whisker plots illustrating Ct values) and normalized fluorescence (right axis, bar graphs showing ΔR values). The best probe (P1.2) is shown in yellow, while other probes are shown in gray. (**B**) Plot shows the performance of single and dual probes for IBV on amplification (left axis, whisker plots illustrating Ct values) and normalized fluorescence (right axis, bar graphs showing ΔR values). The best probe (P2.2) is shown in green, while other probes are shown in gray. (**C**, **D**) Graphs depict the analytical sensitivity of the multiplexed SARS-CoV-2 E, IAV PB1, and RNase P assay (**C**) and multiplexed SARS-CoV-2 RdRP, IBV PA, and RNase P assay (**D**) in the rTEST COVID-19/FLU qPCR kit. (**E**-**H**) Assessment of assay inhibition due to reagent consumption using 4-fold dilutions of samples containing all templates starting at a high viral load (5000x LoD, 10,240 copies/reaction) on cycle threshold (**E**, **F**) and normalized fluorescent intensity (**G**, **H**) of the multiplexed E, IAV, and RNase P assay (**E**, **G**) and the multiplexed RdRP, IBV, and RNase P assay (**F**, **H).** The dotted line at Ct 40 (**C** and **D**) serves as a threshold after which amplification is considered invalid. Ct, cycle threshold; E, envelope gene; IAV, influenza A; IBV, influenza B; PA, polymerase acidic protein; PB1, polymerase basic 1 protein; ND, not detected within 45 cycles; NTC, no template control; RdRP, RNA-dependent RNA polymerase; ΔR, normalized fluorescent intensity.

**Supplementary Table S1. Sequences of all primers and probes that were used during optimization of SARS-CoV-2, IAV and IBV detection**

| **Oligo name** | **Sequence (5´- 3´)** | **Tm (°C)** |
| --- | --- | --- |
| **E gene** | | |
| E_Sarbeco_F1 | ACAGGTACGTTAATAGTTAATAGCGT | 53.8 |
| E_Sarbeco_F2 | CTCATTCGTTTCGGAAGAGACAGG | 57.4 |
| E_Sarbeco_F3 | GTACTCATTCGTTTCGGAAGAGACAG | 57.0 |
| E_Sarbeco_F4 | CGTTTCGGAAGAGACAGGTACG | 57.4 |
| E_Sarbeco_F5 | TGTACTCATTCGTTTCGGAAGAGACA | 57.7 |
| E_Sarbeco_F6 | TGTACTCATTCGTTTCGGAAGAGACAG | 58.1 |
| E_Sarbeco_F7 | A**t**G**t**ACTCATTCGTTTCGGAAGA | 57.8 |
| E_Sarbeco_F8 | A**t**G**t**ACTCATTCGTTTCGGAAGAC | 58.5 |
| E_Sarbeco_F9 | ATG**t**AC**t**CA**t**TCGTTTCGGA | 56.7 |
| E_Sarbeco_F10 | A**t**G**t**AC**t**CATTCGTTTCGGA | 57.0 |
| E_Sarbeco_F11 | ATG**t**ACTCA**t**TCGTT**t**CGGA | 57.1 |
| E_Sarbeco_F12 | A**t**GTACTCAT**t**CGTT**t**CGGA | 57.1 |
| E_Sarbeco_F13 | ATG**t**ACTCA**t**TCGT**t**TCGGA | 56.9 |
| E_Sarbeco_R2 | ATATTGCAGCAGTACGCACACA | 57.2 |
| E_Sarbeco_P1 | FAM/HEX/YY-ACACTAGCCATCCTTACTGCGCTTCG- BHQ1/BHQ2 | 62.3 |
| E_Sarbeco_P2 | FAM/HEX/YY-TAGCGTACTTCTTTTTCTTGCTTTCGTGGT-BHQ1/BHQ2 | 60.1 |
| E_Sarbeco_P1rev | FAM-CGAAGCGCAGTAAGGATGGCTAGTGT-BHQ1 | 62.3 |
| E_Sarbeco_P3 | FAM-TAGCGTACTTCTT**t**TTCTTGCTTTCGTGGT-BHQ1 | 61.3 |
| E_Sarbeco_P4 | FAM-TAGCGTACTTCT**t**T**t**TCTTGCTTTCGTGGT-BHQ1 | 62.5 |
| **RdRP gene** | | |
| RdRP_SARSr-F2 | GTGAAATGGTCATGTGTGGCGG | 59.0 |
| RdRP_Delta-F2 | GTGAAATGGTCATGTGTGGC**A**G | 56.8 |
| RdRP_SARSr-R2 | CGTGACAGCTTGACAAATGTTAAAAAC | 55.7 |
| RdRP_SARSr-R4 | CGTGACAGCTTGACAAATGT**t**AAAAAC | 57.3 |
| RdRP_SARSr-R5 | CGTGACAGCTTGACAAA**t**GT**t**AAAAAC | 58.8 |
| RdRP_SARSr-P2 | FAM/HEX/YY-CAGGTGGAACCTCATCAGGAGATGC-BHQ1/BHQ2 | 61.0 |
| RdRP_SARSr-P3 | FAM-CAGGTGGAACCT-BHQ1-CATCAGGAGATGC-BHQ1 | 61.0 |
| RdRP_SARSr-P4 | FAM-GTGGAACCTCATCAGGAGATGCCAC-BHQ1 | 61.2 |
| RdRP_SARSr-P5 | FAM-GTGGAACCT-BHQ1-CATCAGGAGATGCCAC-BHQ1 | 61.2 |
| RdRP_SARSr-P6 | FAM-GGAACCTCATCAGGAGATGCCACAAC-BHQ1 | 61.2 |
| RdRP_SARSr-P7 | FAM-GGAACCTCAT-BHQ1-CAGGAGATGCCACAAC-BHQ1 | 61.2 |
| RdRP_SARSr-P8 | FAM/HEX/YY-TCAGGAGATGCCACAACTGCTTATGC-BHQ1/BHQ2 | 61.0 |
| RdRP_SARSr-P9 | FAM-TCAGGAGAT-BHQ1-GCCACAACTGCTTATGC-BHQ1 | 61.0 |
| **IAV** | | |
| IAV-F1.1 | TTCTAGCATGGTGGAGGCCAT | 58.5 |
| IAV-F1.2 | ATTTCTAGCATGGTGGAGGCC | 57.3 |
| IAV-F2.1 | AATCCCCTGAATCCCTTTGT | 53.9 |
| IAV-F2.2 | A**a**TCCCCTGA**a**TCCCTTTGT | 57.0 |
| IAV-F2.3 | AA**t**CCCCTGAA**t**CCCTTTGT | 58.2 |
| IAV-R1.1 | CGTCTGAGTTCTTCAATGGTGG | 55.7 |
| IAV-R1.2 | CCGTCTGAGTTCTTCAATGGTGG | 57.7 |
| IAV-R2.1 | TCTGGCATCAATCCGGGC | 58.1 |
| IAV-Probe1.1 | AGGGCCCGGATTGATGCCAGA | 63.6 |
| IAV-Probe1.2 | FAM/HEX/YY-TCTAGGGCCCGGATTGATGCCA-BHQ1/BHQ2 | 62.8 |
| IAV-Probe1.3 | FAM-AAGAG**t**TCTCTG**a**GATCA**t**GA**a**GATC-BHQ1 | 60.7 |
| **IBV** | | |
| IBV-F2.1 | AGTGGACTCAGGAAAGTGGC | 57.1 |
| IBV-F2.2 | TT**t**AGGAT**t**GGCTCCCTATTTGTG | 58.5 |
| IBV-F2.3 | ACTGTGTTTAGGATTGGCTCCCTA | 57.7 |
| IBV-R2.1 | TCCATTTGTTGCATTGATTGAAGC | 55.0 |
| IBV-R2.2 | TCCAT**t**TG**t**TGCATTGATTGAAGC | 57.7 |
| IBV-Probe2.1 | TCCAAATGAAATGGGGAATGGAAGCT | 59.0 |
| IBV-Probe2.2 | FAM/HEX/YY-TCC**a**AATGAA**a**TGGGGAATGGAAGCT-BHQ1/BHQ2 | 60.8 |
| IBV-Probe2.3 | TGCAGAGTGAATGGCACAAATAAGATCCA | 60.2 |
| IBV-Probe2.4 | TGCAGAGTGAATGGCACAAATAAGATCC | 60.9 |
| IBV-Probe2.5 | ACTGTGTTTAGGATTGGCTCCCTATTTGTG | 60.2 |
| IBV-Probe2.6 | CTG**t**GTTTAGGAT**t**GGCTCCCTA**t**T | 61.5 |
| **RNase P** | | |
| RNAse P Forward | AGATTTGGACCTGCGAGCG | 57.9 |
| RNAse P Reverse | GAGCGGCTGTCTCCACAAGT | 59.8 |
| RNAse P Probe | FAM/HEX/YY/Cy5-TTCTGACCTGAAGGCTCTGCGCG-BHQ1/2/3 | 63.3 |
| Lowercase and bold letters represent LNA-modified nucleotides | | |

Red and bold letters represent mismatched base that was corrected for Delta variant

**Supplementary Table S2. Optimization of one-step RT-qPCR** **thermal profiles and reaction mixtures**

| **Kit** | **Alternative thermal profiles tested** |  | **Tested additives/alterations in reaction mixture composition**  (shown are only components, that were changed during optimisation) |
| --- | --- | --- | --- |
| **vDetect v1.** | 50 °C – 10 min  95 °C – 3 min  45x 95 °C – 5 s, 58 °C – 20 s |  | None tested |
|  | **50 °C – 10 min**  **95 °C – 3 min**  **45x 95 °C – 5 s, 60 °C – 20 s** |  |  |
| **vDetect v2.** | **50 °C – 30 min**  **95 °C – 3 min**  **45x 95 °C – 5 s, 60 °C – 20 s** |  | RT/RNase Block: 2 µl |
|  | 50 °C – 10 min  95 °C – 3 min  45x 95 °C – 5 s, 60 °C – 20 s |  |  |
|  | 50 °C – 10 min  97 °C – 3 min  45x 95 °C – 5 s, 60 °C – 20 s |  |  |
| **rTEST** | **55 °C – 10 min**  **95 °C – 10 min**  **45x 95 °C – 15 s, 60 °C – 30 s** |  | 1.5x One-step Probe CoV Mix (ROX) |
|  | 55 °C – 10 min  95 °C – 10 min  45x 95 °C – 10 s, 60 °C – 20 s |  |  |
|  | 55 °C – 20 min  95 °C – 10 min  45x 95 °C – 15 s, 60 °C – 30 s |  |  |
|  | 48 °C – 30 min  95 °C – 10 min  45x 95 °C – 15 s, 60 °C – 30 s |  |  |
| **rTEST Allplex** | 55 °C – 10 min  95 °C – 10 min  45x 95 °C – 25 s, 60 °C – 30 s |  | 80 ng ET SSB  150 ng ET SSB  1.5 mM MgCl_2_  1.5 mM MgCl_2_ + 80 ng ET SSB  200 µM dNTP  200 µM dNTP + 80 ng SSB  200 µM dNTP + 1.5 mM MgCl_2_  400 µM dNTP  400 µM dNTP + 1.5 mM MgCl_2_ |
|  | 55 °C – 10 min  95 °C – 10 min  45x 95 °C – 20 s, 60 °C – 20 s |  |  |
|  | **55 °C – 10 min**  **95 °C – 10 min**  **45x 95 °C – 15 s, 60 °C – 30 s** |  |  |
|  | 55 °C – 10 min  95 °C – 10 min  45x 95 °C – 10 s, 60 °C – 30 s |  |  |
| **rTEST Rapid** | 50 °C – 20 min  95 °C – 10 min  45x 95 °C – 3 s, 60 °C – 10 s |  | None tested |
|  | 50 °C – 20 min  95 °C – 10 min  45x 95 °C – 1 s, 60 °C – 5 s |  |  |
|  | **50 °C – 15 min**  **95 °C – 10 min**  **45x 95 °C – 1 s, 60 °C – 5 s** |  |  |
|  | 50 °C – 15 min  95 °C – 10 min  45x 95 °C – 1 s, 62 °C – 5 s |  |  |
|  | 50 °C – 10 min  95 °C – 10 min  45x 95 °C – 1 s, 60 °C – 5 s |  |  |

**Supplementary Table S3. Cross-reactivity (specificity) testing**

| **Organism** | **vDetect v1** | **rTEST COVID-19** | **rTEST COVID-19/FLU** | | |
| --- | --- | --- | --- | --- | --- |
|  | **E/RdRP** | **E/RdRP** | **E/RdRP** | **IAV** | **IBV** |
| **HCoV-Nl63** | 0/3 | 0/3 | 0/3 | 0/3 | 0/3 |
| **HCoV-229E** | 0/3 | 0/3 | 0/3 | 0/3 | 0/3 |
| **HCoV-OC43** | 0/3 | 0/3 | 0/3 | 0/3 | 0/3 |
| **MERS-CoV**  **(EVAg)** | 0/3 | 0/3 | 0/3 | 0/3 | 0/3 |
| **MERS-CoV (Vircell)** | 0/3 | 0/3 | 0/3 | 0/3 | 0/3 |
| **SARS-CoV HKU39849 (EVAg)** | 0/3 | 0/3 | 0/3 | 0/3 | 0/3 |
| **SARS-CoV (Vircell)** | 0/3 | 0/3 | 0/3 | 0/3 | 0/3 |
| **Influenza A H1N1**  **(Brisbane/59/2007)** | — | 0/3 | 0/3 | 0/3 | 0/3 |
| **Novel Influenza A H1N1 (/California/07/2009)** | — | 0/3 | 0/3 | 3/3 | 0/3 |
| **Influenza A H3N2**  **(Perth/16/2009)** | — | 0/3 | 0/3 | 3/3 | 0/3 |
| **Influenza A H5N1 (Viet Nam/1194/2004 x Puerto Rico/8/1934)** | — | 0/3 | 0/3 | 0/3 | 0/3 |
| **Novel Influenza B (/Brisbane/60/2008)** | — | 0/3 | 0/3 | 0/3 | 3/3 |
| **Human parainfluenza 1** | — | 0/3 | 0/3 | 0/3 | 0/3 |
| **Respiratory syncytial virus (subtype A)** | — | 0/3 | 0/3 | 0/3 | 0/3 |
| **Human rhinovirus (B14)** | — | 0/3 | 0/3 | 0/3 | 0/3 |
| **SARS-CoV-2 positive control** | 3/3 | 3/3 | 3/3 | 0/3 | 0/3 |
| **BMC1** | 2/2 | — | — | — | — |
| **PC BMC 5** | — | 3/3 | 3/3 | — | — |
| **JRC** | 2/2 | — | — | — | — |
| **Negative control** | 0/20 | 0/3 | 0/3 | 0/3 | 0/3 |

**Supplementary Table S4. Clinical performance of vDetect v.1 COVID-19 RT-qPCR kit**

|  | **Index test (Charité, Promega)** | | **vDetect COVID-19 RT-qPCR kit laboratory 1** | | **vDetect COVID-19 RT-qPCR kit laboratory 2** | |
| --- | --- | --- | --- | --- | --- | --- |
|  | **E gene** | **RdRP gene** | **E gene** | **RdRP gene** | **E gene** | **RdRP gene** |
| **Number of correctly identified positive samples** | 38 | 38 | 38 | 38 | 38 | 38 |
| **Number of false positive samples** | 2 | 0 | 0 | 0 | 0 | 0 |
| **Number of correctly identified negative samples** | 52 | 54 | 54 | 54 | 54 | 54 |
| **Number of false negative samples** | 0 | 0 | 0 | 0 | 0 | 0 |

**Supplementary Table S5. Clinical performance of rTEST COVID-19 qPCR kit**

|  | **Index test (vDetect v1)** | | **rTEST COVID-19 qPCR kit** | | | | |  |
| --- | --- | --- | --- | --- | --- | --- | --- | --- |
|  |  |  |  |  |  |  |  |  |
|  | **E gene** | **RdRP gene** | **E gene** | | **RdRP gene** | | **RNase P gene** |  |
| **Number of correctly identified positive samples** | 37 | 33 | 38 | | 38 | | 38 |  |
| **Number of false positive samples** | 0 | 0 | 0 | | 0 | | 0 |  |
| **Number of correctly identified negative samples** | 54 | 54 | 54 | | 54 | | 54 |  |
| **Number of false negative samples** | 1 | 5 | 0 | | 0 | | 0 |  |
|  | | | |  | |  | |  |

**Supplementary Table S6. Clinical performance of rTEST COVID-19 qPCR Multiplex and Allplex kits**

|  | **Index test (vDetect v1)** | | | **rTEST COVID-19 qPCR Multiplex and Allplex kits*** | | |  |
| --- | --- | --- | --- | --- | --- | --- | --- |
|  |  |  |  |  |  |  |  |
|  | **E gene** | **RdRP gene** | **RNase P gene** | **E gene** | **RdRP gene** | **RNase P gene** |  |
| **Number of correctly identified positive samples** | 30 | 30 | 30 | 30 | 30 | 30 |  |
| **Number of false positive samples** | 0 | 0 | 0 | 0 | 0 | 0 |  |
| **Number of correctly identified negative samples** | 30 | 30 | 30 | 30 | 30 | 30 |  |
| **Number of false negative samples** | 0 | 0 | 0 | 0 | 0 | 0 |  |
| *The results of rTEST COVID-19 qPCR Multiplex and Allplex kits are combined because they were compared to the same index test and there were no differences in clinical sensitivity or specificity | | | | | | |  |

**Supplementary Table S7. Clinical performance of rTEST COVID-19 qPCR Rapid kit**

|  | **Index test (rTEST Allplex)** | | | **rTEST COVID-19 qPCR Rapid kit** | | |  |
| --- | --- | --- | --- | --- | --- | --- | --- |
|  |  |  |  |  |  |  |  |
|  | **E gene** | **RdRP gene** | **RNase P gene** | **E gene** | **RdRP gene** | **RNase P gene** |  |
| **Number of correctly identified positive samples** | 105 | 105 | 105 | 105 | 103 | 105 |  |
| **Number of false positive samples** | 0 | 0 | 0 | 0 | 0 | 0 |  |
| **Number of correctly identified negative samples** | 94 | 94 | 94 | 94 | 94 | 94 |  |
| **Number of false negative samples** | 0 | 0 | 0 | 0 | 0 | 0 |  |
| **Number of inconclusive samples** | 0 | 0 | 0 | 0 | 2 | 0 |  |

**Supplementary Table S8. Clinical performance of rTEST COVID-19/FLU qPCR kit**

|  | **Index test** | | | **rTEST COVID-19/FLU qPCR kit** | | |  |
| --- | --- | --- | --- | --- | --- | --- | --- |
|  |  |  |  |  |  |  |  |
|  | **E gene** | **RdRP gene** | **RNase P gene** | **E gene** | **RdRP gene** | **RNase P gene** |  |
| **Number of correctly identified positive samples** | 37 | 33 | 33 | 38 | 38 | 38 |  |
| **Number of false positive samples** | 0 | 0 | 0 | 0 | 0 | 0 |  |
| **Number of correctly identified negative samples** | 54 | 54 | 54 | 54 | 54 | 54 |  |
| **Number of false negative samples** | 1 | 5 | 5 | 0 | 0 | 0 |  |

|  | **Index test** | | | **rTEST COVID-19/FLU qPCR kit** | | |  |
| --- | --- | --- | --- | --- | --- | --- | --- |
|  |  |  |  |  |  |  |  |
|  | **IAV PB1 gene** | **IBV PA gene** | **RNase P gene** | **IAV PB1 gene** | **IBV PA gene** | **RNase P gene** |  |
| **Number of correctly identified positive samples** | 52 | 37 | 89 | 52 | 37 | 89 |  |
| **Number of false positive samples** | 0 | 0 | 0 | 0 | 0 | 0 |  |
| **Number of correctly identified negative samples** | 1 | 1 | 2 | 1 | 1 | 2 |  |
| **Number of false negative samples** | 0 | 0 | 0 | 0 | 0 | 0 |  |
